# School-based sexual health education interventions to prevent STI/HIV in sub-Saharan Africa: a systematic review and meta-analysis

**DOI:** 10.1186/s12889-016-3715-4

**Published:** 2016-10-10

**Authors:** A. Sadiq Sani, Charles Abraham, Sarah Denford, Susan Ball

**Affiliations:** 1 Psychology Applied To Health, University of Exeter Medical School, College House, St Lukes Campus, Exeter, EX1 2 LU UK; 2 NIHR CLAHRC South West Peninsula (PenCLAHRC), University of Exeter Medical School, St Luke’s Campus, Exeter, EX1 2 LU UK

**Keywords:** Systematic review, School-based sexual health education, Sub-Saharan Africa, HIV/STI prevention

## Abstract

**Background:**

School-based sexual health education has the potential to provide an inclusive and comprehensive approach to promoting sexual health among young people. We reviewed evaluations of school-based sexual health education interventions in sub-Saharan Africa to assess effectiveness in reducing sexually transmitted infections and promoting condom use.

**Methods:**

We searched ten electronic databases, hand-searched key journals, and reference lists of included articles for potential studies. Data were extracted on outcomes, intervention characteristics, methods and study characteristics indicative of methodological quality. Where possible, data were synthesized using random effect meta-analysis. Intervention features found predominantly in effective interventions were noted.

**Results:**

The initial search retrieved 21634 potentially relevant citations. Of these, 51 papers reporting on 31 interventions were included. No evaluation reported statistically significant effects on the incidence or prevalence of Human Immunodeficiency Virus and Herpes Simplex Virus 2 infections. However, intervention participants reported statistically significant greater condom use in both randomised controlled trials and non-randomised trials for short (less than 6 months) follow-up periods (*OR* = 1.62, 95 % *CI* = 1.03–2.55 and *OR* = 2.88, 95 % *CI* = 1.41–5.90 respectively). For intermediate (6–10 months) and long-term (more than 10 months) follow-up periods, the effect was statistically significant (*OR* = 1.40, 95 % *CI* = 1.16–1.68) and marginally significant (*OR* = 1.22, 95 % *CI* = 0.99–1.50) among the randomised trials respectively. Only 12 of the 31 interventions reported implementation details, out of which seven reported on fidelity.

**Conclusion:**

School-based sexual health education has the potential to promote condom use among young people in sub-Saharan Africa. However, further work is needed to develop and evaluate interventions that have measurable effects on sexually transmitted infections.

**Electronic supplementary material:**

The online version of this article (doi:10.1186/s12889-016-3715-4) contains supplementary material, which is available to authorized users.

## Background

Worldwide, more than one million cases of sexually transmitted infections (STIs) occur daily and 500 million people live with curable STIs including Chlamydia, Gonorrhoea, Syphilis and Trichomoniasis [1]. The burden of STIs is high in sub-Saharan Africa (sSA) with an incidence rate of 241 per 1000 among adults age 15–49, one of the highest in the world [2]. Approximately 70 % of those living with Human Immunodeficiency Virus (HIV) worldwide, and 80 % of infected women aged 15–24, live in sSA where one in six adolescent deaths is attributed to HIV [3, 4]. Approximately half of new HIV infections occur in individuals aged 15–24 [5] and more than 90 % are sexually transmitted. Thus, sexually active young people in sSA, including young women, are at high risk of HIV infection.

Several types of interventions have been employed to reduce the vulnerability of adolescents to STIs, including HIV. These include: preventive education in schools; services delivered in youth centres, including condom distribution; adolescent-friendly health centres that encourage utilisation of prevention services; school-based health services; conditional cash transfers to encourage young people to remain in school or to avoid risky sexual behaviours; various community-based interventions; and unconditional cash transfers [6]. School-based sexual health education (SBSHE) is arguably the most inclusive and potentially comprehensive of these approaches and has the potential to effectively promote population-level sexual health among adolescents and young adults [7], so reducing the spread of STIs, including HIV [8]. Mavedzenge et al. [6] found high quality evidence for effectiveness of SBSHE in relation to a number of STI-related outcomes from evaluations worldwide and recommended such interventions be implemented widely. However, evaluations of SBSHE interventions in sSA have provided mixed findings in relation to reduction of self-reported unprotected sexual intercourse and surprisingly, none has provided evidence of reduction of STI incidence or prevalence [8–10]. In this review, SBSHE was defined as any intervention delivered wholly or partially in a school setting aiming to reduce risky sexual behaviours, STIs and unplanned pregnancy, and promote positive sexual health.

Four reviews of evaluations of SBSHE interventions in Africa were found [8–10]. None assessed effects of interventions on STI incidence or prevalence. A review by Kaaya et al. [9] included 11 interventions and concluded that most studies reported effects on knowledge, attitudes and communication but fewer reported effects on self-reported onset of sexual intercourse, frequency of sexual intercourse and number of sexual partners. Similarly, a review by Gallant and Maticka-Tyndale [8] also including 11 interventions and concluded that knowledge and attitudes are easier to change than behaviours among African youths. These reviewers recommended that intervention design should be grounded in theorized accounts of behaviour change mechanisms and be directly relevant to local culture. A third review by Paul-Ebhohimhen et al. [10], including 10 evaluations, also concluded that SBSHE interventions had stronger effects on sexual health knowledge and attitudes than on behaviour patterns. More optimistically, a review of seven interventions for Nigerian students found changes in self-reported sexual behaviour patterns including delaying sexual debut, increasing condom and other contraceptives use and reducing frequency of sexual activity [11]. Other reviews include SBSHE interventions [5, 12–26] but draw no conclusions about SBSHE in sSA.

### The present study

This review extends previous reviews of effectiveness of SBSHE interventions in sSA [8–10] in four key respects. First, we include an updated and more comprehensive set of experimental evaluations. Second, we assess intervention effects on reduction of STIs indexed by biological markers. Third, we explore intervention characteristics that may differentiate between effective and ineffective interventions. Fourth, we examine assessment of implementation fidelity.

The review addressed three research questions:

1. How effective are school-based sexual health education interventions in sub-Saharan Africa in promoting condom use and preventing sexually transmitted infections?
2. What characterizes effective school-based sexual health education interventions implemented in sub-Saharan Africa?
3. Are school-based sexual health education interventions implemented with fidelity in sub-Saharan Africa?

## Methods

This review was conducted according to a protocol [see Additional file 1, for the review protocol], and reported in accordance to PRISMA statement [27].

### Inclusion criteria

Studies were included if they met the following criteria:

- i. Published in English before March 2016.
- ii. The study was a randomised control trial (RCT) or quasi-experimental (non-randomised trials and before-and-after studies with comparison groups).
- iii. More than 80 % of participants were below the age of 25 years. A 25 year cut-off was applied because age of school enrolment varies considerably across sub-Saharan Africa, particularly in the rural areas. Hence, it is not uncommon to find older students in primary or secondary schools [28].
- iv. The study evaluated a school-based sexual health education intervention delivered in sub-Saharan African schools. The intervention could be delivered completely in school or include components delivered to school students outside school and/or outside school hours.
- v. The dependent measure was self-reported condom use and/or levels of STIs.

### Exclusion criteria

Studies were excluded for the following reasons:

- i. They employed no comparison or control group.
- ii. They employed a comparison group that received sexual health education other than the usual curriculum.
- iii. They were delivered in universities.
- iv. Twenty percent or more of the participants were aged 25 years and above.
- v. Knowledge, attitudes and behavioural intentions were the only outcomes reported.

### Search strategy

Ten electronic databases including Medline, PsycInfo, EMBASE, CINAHL, Web of Knowledge, The Cochrane Library, British Education Index/EBSCOhost, Australian Education Index/ProQuest, Education Research Complete/EBSCOhost and ERIC/ProQuest were searched in February 2015 (see Additional file 2: Table S2, which contains search strategy for Medline that was modified and used in other databases). One new inclusion was identified in an updated search run in March 2016. We also searched the table of contents of Journal of AIDs, AIDs Care, AIDs and Behaviour, AIDs Education and Prevention, Journal of Adolescent Health, and Journal of Youth and Adolescence for relevant studies. Reference lists of similar reviews and included studies were also searched in an iterative fashion until no new article was found.

### Study selection

Titles and abstracts of the 21,634 identified studies were screened by the primary reviewer (SAS) with a random selection (*n* = 500, 2.3 %) screened by a second reviewer (SD). Full texts of articles that passed the title-abstract stage were obtained for full text screening. All the full text articles were screened by SAS and 20 % (*n* = 53) randomly selected were screened independently by SD. Gwet’s [29] AC1 statistic was used to assess the inter-rater reliability at each stage of the screening and any disagreement was resolved through discussion. The opinion of a third reviewer (CA) was sought when, exceptionally, two reviewers (SAS and SD) were unsure how to resolve disagreements.

### Data extraction

We extracted data relevant to the review questions, including study design, descriptions of the interventions, theories informing intervention design, features of effective interventions, descriptions of implementation and outcomes categorised by length of follow-up. The data extraction form is available as a Additional file 3: Table S3. Where more than one article described the same intervention, data were extracted from all papers. Data was extracted by the primary reviewer (SAS) and independently by a second reviewer (SD) to check accuracy. Furthermore, a statistician (SB) also extracted quantitative outcomes data included in meta-analysis independently.

### Quality assessment of included studies

The quality of the included studies was assessed using four main dimensions based on recommendations in the Cochrane Collaboration Tool for Assessing Risk of Bias [30], namely selection bias, performance bias, detection bias, and attrition bias of the included studies. The Cochrane Collaboration Tool was used to assess the quality of included interventions because it is a domain-based evaluation that gives critical assessment of each domain (dimension) in which bias may arise [30]. It has the advantage of encouraging users to tailor how they assess studies and so adds to transparency unlike some other methodology assessment checklists (e.g., Jadad [31]). Selection bias for non-RCTs was assessed by determining selection bias due to confounding as recommended in the Cochrane Collaboration Handbook [30]. Random sequence generation or allocation concealment (or bias due to confounding for non-RCTs) and incomplete outcome data were considered critical for assessing the quality of studies in this review. The critical dimensions were used to score the overall risk of bias of the included studies. Random sequence generation and allocation concealment were scored as one dimension assessing selection bias. A score of two was given for ‘high’, one for ‘unclear’ and zero for ‘low’ risk of bias. Therefore, an intervention can have an overall score ranging from zero to four. An article with a total score of 3 or 4 was assessed as ‘high’, 2 as ‘moderate’ and 0 or 1 as ‘low’ risk of bias.

### Data analysis

Review Manager 5.3 software [32] was used to undertake meta-analyses identifying intervention effectiveness in relation to increased condom use and reduced HSV2 infections using outcome measures closest to the median follow-up period. Separate analyses for condom use were conducted dividing evaluations into those with short (less than 6 months), intermediate (6–10 months, based on a median of 8 months) and long-term follow-up (more than 10 months). Random-effects method of meta-analysis that is based on inverse-variance technique that adjusts for varying study weights and heterogeneity was employed [30] because of variability in trial size and intervention content, intensity and duration. Whenever available, adjusted (for baseline) rather than crude odds ratios (*OR*) were used in the analyses. Heterogeneity across estimates was quantified using the I-squared statistic (*I* ^2^) and the *p*-value from the chi-squared test for heterogeneity was used to quantify evidence against homogeneity [30]. We did not include studies in meta-analysis if heterogeneity was high (*I* ^2^ of 75 % and above). Those studies that provided insufficient data to include in the meta-analyses were reported descriptively.

We also conducted sensitivity, or sub group, analyses to assess the effects of two study characteristics on effectiveness, namely, (i) the measure of condom use employed (condom use at last sex versus other measures) and (ii) use of crude versus adjusted odds ratios.

### Quality of evidence

We used “Grading of Recommendations Assessment, Development and Evaluation” (GRADE) [33] to assess the overall quality of evidence reported in studies included in meta-analyses. This approach provides a structured and transparent way of developing and presenting results summaries that are easy to understand [33]. Five criteria were used in grading the evidence including limitations of design (risk of bias), inconsistency (heterogeneity), indirectness, imprecision, and reporting or publication bias. For limitations of design (risk of bias), the quality was downgraded if most of the studies were of high risk of bias as assessed with the Cochrane Collaboration Tool. For inconsistency, unexplained heterogeneity indicated by *I* ^2^ of more than 75 % was used to downgrade the quality of evidence for this criterion. Indirectness was assessed by determining how closely the interventions, participants and measures of outcome of interest were similar across studies. A relative risk reduction or increase of greater than 25 % (±0.25) as suggested by GRADE was used to downgrade the quality of evidence for imprecision. Finally, visual inspection of asymmetry of funnel plots was used to detect the possibility of publication bias, and quality was downgraded if asymmetry was observed. These assessments were undertaken using GRADEpro software [34] and a summary of findings table (SoF) generated. The overall quality of each outcome was graded as ‘high’, ‘moderate’, ‘low’ and ‘very low’. These are interpreted as ‘very confident’, ‘moderately confident’, ‘limited confidence’ and ‘very low confidence’ that the true effect lies close to the estimated effect respectively [33].

### Features of effective interventions

Intervention design and implementation characteristics associated with effectiveness have been identified in previous reviews. Design related features include: needs assessment with the intended participants and involving key stakeholders in the design or development process of the intervention [5, 10]; adapting the intervention or curriculum from other evaluated interventions [5]; basing the intervention on behavioural change theory [9]; and providing the participants with skills that help reduce risky sexual behaviours [10). Implementation characteristics include: provision of adolescent-friendly health services [5]; distribution of condoms [5]; extending activities to the community outside the school environment [5]; training of facilitators; and fidelity of delivery [10]. Intervention descriptions in the current review were coded for inclusion of these features. We classified interventions as “interventions with evidence of benefit” or “interventions without evidence of benefit”. “Evidence of benefit” was identified as a statistically significant increase condom use or less prevalence/incidence of STI at any follow-up among any sub-group of the participants (e.g., among sexually active participants). The frequency of occurrence of potentially effectiveness enhancing features among the interventions with evidence of benefit and those without evidence of benefit was then determined.

An intervention was regarded to have been delivered with fidelity if the authors reported that the intervention was delivered as intended. This includes how “faithful” components, content, and activities of the intervention were delivered as designed. It also includes frequency and duration of exposure (intensity) of the intervention.

## Results

### Selection and description of studies

**Fig. 1:**
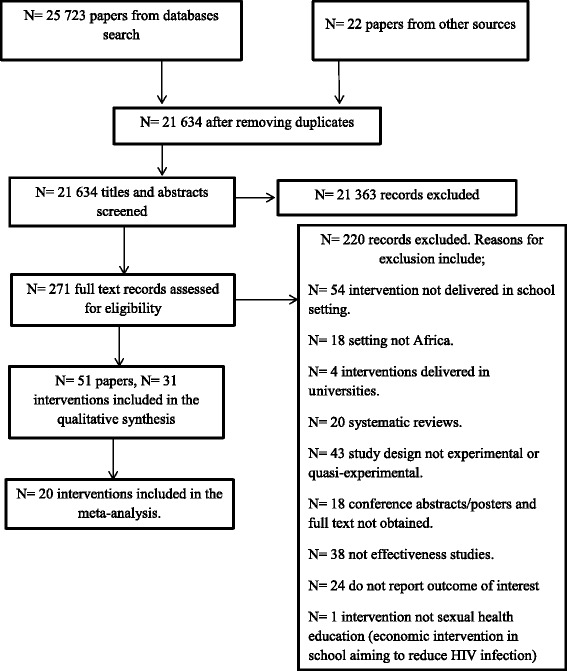
Studies selection flow diagram

We obtained 21,634 papers after removing duplicates (Fig. 1), out of which 271 were selected after screening through titles and abstracts (AC1 = 0.98). After full-text screening two reviewers (SAS and SD) independently screened and agreed (100 % agreement, AC1 = 1.0) on inclusion of 51 papers, reporting on 31 interventions. The *Mema Kwa Vijana* (MkV) intervention was reported in six included papers [35–40], however, Ross et al. [35] is cited henceforth when referring to this intervention because most data were extracted from that report. Similarly, other interventions reported in more than one paper include: (i) Primary School Action for Better Health (PSABH) [41, 42]. (ii) HealthWise South Africa [43–46]. (iii) Promoting Sexual and Reproductive Health, School-based HIV/AIDS Intervention in Sub-Saharan Africa (SATZ) [47–50]. (iv) HIV Prevention Intervention for Rural Youth (HP4RY) [51–54]. (v)‘Let Us Protect Our Future’ South Africa [55–57]. (vi) *The Regai Dzive Shiri Project* [58–61]. Subsequently, key papers ([41, 43, 47, 51, 55, 58] respectively) are cited when referring to these interventions.

Seventeen of the evaluations were RCTs and 14 used quasi-experimental designs. Twenty-six of the interventions were delivered in secondary or high schools (84 %), four (13 %) in primary or elementary schools and SATZ intervention in both primary and secondary schools (3 %). Four interventions [35, 51, 58, 62] included out-of-school and community activities, some involving health centres. The MkV intervention [35] had four components namely, a teacher- and peer-led in-school programme; provision of youth-friendly health services; condom promotion and distribution; and community mobilisation activities. The HP4RY intervention [51] had two components, a teacher delivered school programme and a community programme delivered by young people. The *Regai Dzive Shiri Project* [58], had three components, teacher-delivered school activities, community activities targeting parents and other community members, and provision of reproductive health services. The intervention by Brieger et al. [62] was a peer-led activity involving in-school activities as well as visits to, clinics and community centres activities. All interventions included in this review had both female and male participants, and participants were 9–30 years old [55, 63]. The number of participants varied from 24 [64] to 13814 [35], with a total number 70201 across all included evaluations. The median follow-up period for condom use was 8 months and 54 months for the biological outcomes. See Additional file 4: Table S4, which provides lists of excluded studies with reasons for the exclusion and Table 1, which provides the characteristics of the included studies. Intervention descriptions included in Table 1 are summaries of those provided in the papers describing included interventions. All studies reported on ethical approval and/or received informed consent from the participants.

**Table 1:** Characteristics of Included Studies

STUDY (lead author surname and date)	SETTING (LEVEL & COUNTRY)	STUDY DESIGN	PARTICIPANTS’ AGE (YEARS) & NUMBER	INTERVENTION	COMPARISON	LENGTH (S) OF FOLLOW UP
Aderibigbe 2008	Secondary schools, Nigeria	Quasi-experimental	Age range: 10–19 Number: 521	*Objectives:* to reduce risky sexual behaviours. *Content:* topics on HIV/AIDs, sexual behaviours including condom use and risks of exchange of sex for gifts were covered. *Activities:* health education sessions consisting of lectures, film shows, and distribution of information, education and communication (IEC) materials. *Dose/frequency/duration:* not reported (NR) *Theory used*: NR	No intervention	3 months
Agha 2004	Secondary schools, Zambia	Quasi-experimental	Age range: 14–23 Number: 416	*Objectives:* to increase knowledge, normative belief and self-risk perception of contracting HIV. *Content:* curriculum provided factual information on HIV, modes of transmissions, impact of the infection on immunity, high risk associated with anal sex and other non-sexual modes of transmission of HIV. It also contained information on abstinence, correct and consistent use of condom. *Activities:* peer led discussions, drama skits and distribution of educational leaflets. *Dose/frequency/duration:* one session of 1-h-and-45-min duration. *Theory used:* NR	1-h-long, peer-led water purification intervention.	1 week and 6 months
Ajuwon 2007	Secondary schools, Nigeria	Quasi-experimental	Age range: 10–25 Number: 494	*Objectives:* to improve secondary school students’ sexual health knowledge, attitudes, perceived self-efficacy and sexual practices. *Content:* information on aspects of reproductive health, STI/HIV/AIDS and condoms were covered*.* *Activities:* teacher instructions, presentations, rotational talks, health quiz competitions, drama presentations, counselling of students and distribution of condoms with other educational materials. *Dose/frequency/duration:* nine months (i.e., one academic session) *Theory used:* NR	No intervention	Immediate
Arnold 2012	Secondary schools, Nigeria	Cluster Randomised Controlled Trial	Age range: greater than 11 to less than 17 Number: 2589	*Objectives:* to reduce vulnerability of youths to HIV infection. *Content:* the school curriculum included topics on human development, HIV infection, sexual behaviours, personal skills, relationships, and society and culture. *Activities:* the participants received family life and HIV education programme in schools as well as community interventions from youths trained in working with youths and adults. *Dose/frequency/duration:* the school curriculum was delivered over 3 years of junior secondary schools. *Theory used:* Social Ecology Theory, Social Scripting Theory and AIDs Competent Community Model	No intervention while waiting for delayed intervention.	12 months and 18 months
Atwood 2012	Elementary or middle school, Liberia	Randomised Controlled Trial	Mean age: 16.3 Number: 812	*Objectives:* to promote attitudes and skills for safer sex. *Content:* an eight-module programme designed to promote attitudes and skills for safer sex. These include positive attitudes towards condom use, skills to negotiate condom use, refuse sex and use condoms effectively. *Activities:* NR *Dose/frequency/duration:* one module per week over eight weeks. *Theory used:* Social Cognitive Theory and Theory of Reasoned Action.	General health curriculum intervention which includes information on how to prevent malaria, Tuberculosis, worm infestation and HIV/STD knowledge that do not have behavioural theory underpinning and preventive skills.	3 months and 9 months
Brieger 2001	Secondary, post-secondary and out of school youths, Nigeria and Ghana	Quasi-experimental	Age: Adults below 25 years of age Number: 1784	*Objectives:* to improve knowledge of reproductive health, and promote safe sex behaviours with contraceptive use among sexually active adolescent. *Content:* information on sexuality and reproductive health, safer sex behaviours and contraceptives was provided. *Activities:* peer counselling, youth involvement in information education and communication materials development, drama, and provision of contraceptives. Other activities include TV, radio, youth centre activities, nurse workshops, clinic visits, youth centre and street campaigns. *Dose/frequency/duration:* 18 months. *Theory used:* NR	No intervention	18 months
Burnett 2011	High schools, Swaziland	Randomised Controlled Trial	Mean age: 17.35 Number: 177	*Objectives:* an American HIV education programme adapted for Swaziland to improve HIV related knowledge, attitudes and safe sexual behaviours including HIV testing. *Content of curriculum:* Topics including “understanding my body”, basics of HIV and STIs, HIV testing, prevention and treatment of HIV, stigma and discrimination for people living with HIV, relationships and assertive behaviours. *Activities:* interactive techniques such as role play and group discussions. *Dose/frequency/duration:* one hour per week for 13 weeks. *Theory used:* Self-efficacy Theory.	No intervention	Immediate
Cowan 2010	Secondary schools & community clinics, Zimbabwe	Cluster Randomised Controlled Trial	Age range: 18–22 Number: 6791	*Objectives:* a community-based multi-component HIV prevention intervention aiming to change adolescents’ social norms. *Content:* Modified version of MkV curriculum (see Ross 2007 below). Also included sessions on self-awareness, communication, self-belief and gender issues. *Activities:* peer educators led intervention; parents and community stakeholders programme to improve health knowledge, communication between parents and youths; and community support for adolescent reproductive health and provision of reproductive health services by nurses and staff working in rural clinics. *Dose/frequency/duration:* NR *Theory used:* Social Learning Theory and The Stages of Change Model.	No intervention	48 months
Cupp 2008	High schools, South Africa	Cluster Randomised Controlled Trial	Age range: 13–18 Number: 1095	*Objectives:* to reduce risky behaviours concerning alcohol and sex. *Content:* 40 % focused on alcohol-related issues, while the other 60 % on reducing risky sexual activity to avoid HIV, other STIs and unwanted pregnancy. *Activities:* role-plays, teachers and peers led group discussions and audio vignettes. *Dose/frequency/duration:* 30–40 min per unit (15 units in total) over 8 weeks. *Theory used:* Social Learning Theory, Theory of Planned Behaviour and Social Inoculation Theory.	Regular Life Orientation curriculum.	4-6 months and 14–18 months
Denison 2012	High schools, Zambia	Quasi-experimental	Age range: 11 to less than 19. Number: 2476	*Objectives:* to increase knowledge, attitudes and protective behaviours related to HIV and reproductive health. *Content:* curriculum addressed life skills and sexual behaviours including abstinence, being faithful and condom use. *Activities:* provision of library materials and counselling from a youth resource centre; volunteer peer educators coordinated extracurricular activities; educational events to communities on specific topics; and workshops to teachers on specific topics as a way of capacity building. *Dose/frequency/duration:* 40 min weekly over 7–9 months. *Theory used:* NR	No intervention	NR
Esere 2008	Secondary schools, Nigeria	Quasi-experimental	Age range: 13–19 Number: 24	*Objectives:* to reduce risky sexual behaviours and improve quality of sexual behaviours among school going adolescents. *Content:* topics on puberty, reproduction, contraception, and negotiation in relationships, including training in assertiveness skills were covered. *Activities:* active learning through small group discussions and games; skills development through role-play; and information dissemination through leaflets. Subjects on puberty, reproduction, contraception and negotiation/assertiveness skills were taught. *Dose/frequency/duration:* one session per week over eight weeks. *Theory used:* NR	No intervention	Immediate
Fawole 1999	Secondary schools, Nigeria	Randomised Controlled Trial	Mean age: 17.6 (intervention group), 17.8 (control group). Number: 450	*Objectives:* to improve knowledge, attitude and sexual risk behaviours of secondary school students. *Content:* the course targeted knowledge, attitudes and sexual behaviours in relations to STIs including HIV. *Activities:* film shows, lectures, stories, role-plays, songs, essays and debates as well as demonstration on how to use a condom. *Dose/frequency/duration:* two hours per session, one session per week over six weeks. *Theory used:* NR	No intervention	6 months
James 2005	Secondary schools, South Africa	Randomised Controlled Trial	Age range: 15 to less than 22 Number: 1168	*Objectives:* to improve knowledge, attitudes, communication and behavioural intentions concerning sexually transmitted infection. *Content:* Laduma print provides the reader with information on sexually transmitted infections and clears any misconception on the issue. It also provides information that will bring about attitudinal change on the participants including safe sex behaviours, self-efficacy and adaptation skills for safe sex behaviour. Condom use for prevention of STI is clearly explained in Laduma. *Activities:* Laduma print was given to participants to read. *Dose/duration/frequency:* once and take averagely one hour to read the print. *Theory Used:* NR	No intervention	3 weeks and 6 weeks
James 2006	Secondary schools, South Africa	Randomised Controlled Trial	Age range: 12-21years Number: 1141	*Objectives:* to improve knowledge of HIV/AIDS and its prevention; safer sex practices and intentions to practice safer sex; and positive attitudes toward condom use and people living with AIDS. *Content:* topics included information about HIV and AIDS, modes of transmission, the immune system, the progression of HIV to AIDS, and how to avoid HIV infection. Knowledge, attitude to condom use and people living with AIDS, gender norms, and perceptions about sexual behaviour were addressed. *Activities:* programs were delivered through combination of different methods including didactic and interactive group work, teaching and role-play guided by a prescribed manual. *Dose/duration/frequency:* one lesson per week over two school terms (20 weeks). *Theory used:* NR	Students in the control group received odd lessons about aspects of HIV and AIDS education in a non-structured format and in some cases celebrated awareness days on the topic.	6 months and 10 months
Jemmott 2015	Primary schools, South Africa	Cluster Randomised Controlled Trial	Age range: 9–18 Number: 1057	*Objectives:* to increase knowledge on HIV risk reduction, sexuality, sexual maturation, sex role and rape myth beliefs, and skills/self-efficacy to negotiate sex. *Content:* topics covered included HIV/STD risk-reduction knowledge; behavioural beliefs that support abstinence and condom use; skills and self-efficacy negotiating abstinence and condom use and to use condoms; and sex-specific modules that addressed sexuality, sexual maturation, appropriate sex roles, and rape myth belief. *Activities:* games, role-playing, group discussions, brainstorming and comic workbooks using series of storylines and characters. *Dose/frequency/duration:* 12 one hour modules (delivered 2 modules per day) over 6 days. *Theory used:* Social Cognitive Theory and Theory of Planned Behaviour.	Health-promotion intervention designed to increase fruit and vegetable consumption and physical activity and decrease cigarette smoking and alcohol use.	3 months, 6 months, 12 months, 42 months and 54 months
Karnell 2006	Secondary schools, South Africa	Quasi-experimental	Median age: 16 Number: 661	*Objectives:* to give facts related to HIV and alcohol; consequence and alternatives to drinking alcohol and having unprotected sex; and techniques to resist drinking and having sex. *Content:* half of the curriculum focused on alcohol related issues, while the remaining half on HIV-related issues. *Activities:* the intervention was delivered as monologues role-play delivered by four fictional teenage characters that served the basis for class discussion and group assignments. *Dose/duration/frequency:* 10 units, 30 min each over 8 weeks. *Theory used:* Social Learning, Social Inoculation and Cognitive Behaviour Theory.	Regular Life Orientation curriculum.	5 months
Mason-Jones 2011	High schools, South Africa	Quasi-experimental	Age range: 15–16 Number: 3934	*Objectives:* to delay sexual debut and increase use of condoms. *Content:* The intervention consisted of a mixture of taught weekly classroom sessions by peer educators following a standard curriculum covering issues on relationships, well-being and sexual health and confidence building. *Activities:* It consists of weekly classroom taught sessions by peer educators trained on issues related to sexual health, confidence building, sexual health and wellbeing. *Dose/frequency/duration:* NR *Theory used:* NR	Comparison schools received their usual Life Orientation programme.	18 months
Mason-Jones 2013	High schools, South Africa	Quasi-experimental	Age range: 15–16 Number: 728	*Objectives:* a high school peer educators training programme to improve safe sexual behaviours and related psychosocial outcomes of the peer educators. *Content:* training included information about sexual and reproductive health including HIV/AIDS and about community services available, learning about leadership, presentation skills, life skills lessons, communication skills, group work and community development. It also included the development of psychosocial skills believed to be protective in reducing risky sexual behaviours such as goal orientation, critical thinking, self-esteem and decision-making. *Activities:* the intervention includes training peers that involves giving information on reproductive health including HIV/AIDs, availability of reproductive health services, life skills, presentation skills, communication skills, group work and community development. *Dose/frequency/duration:* two training sessions (1 h each per month), 11 training sessions (over 3-day camp). *Theory used:* NR	Students from comparison schools received no extra training.	18 months
Mathews 2012	High schools, South Africa and Primary Schools, Tanzania	Cluster Randomised Controlled Trial	Age range: 12–14 Number: 12139	*Objectives:* to reduce young adolescent risky sexual behaviours including delaying sexual debut and promoting condom use. *Content:* topics included self-image and values clarification; personal, social and physical development, sexuality and reproduction; HIV, AIDS, STIs and substance use; condom use; gender roles; skills for protection and safety; intimate partner violence; contraception; sexual decision-making and sexual risk behaviour; sexual risk assessment; myths and misconceptions; healthy lifestyle; and reproductive health rights. *Activities:* teacher led presentations, small group discussions, skills training, small group activities, role-play, condom demonstrations, quiz, drama, song composition and homework to involve parents. *Dose/frequency/duration:* one school semester of approximately 5 months duration and 15–20 school hours. *Theory used:* Attitude-Social Influence Efficacy (ASE) model.	No intervention	6 months and 12–15 months
Maticka-Tyndale 2007	Primary schools, Kenya	Quasi-experimental	Age range: 11–16 Number: 3452	*Objectives:* to provide information on transmission of HIV and skills building to withstand social, cultural or interpersonal pressure to involve in risky sexual behaviours as well as skills to reduce stigma to people living or affected by HIV. *Content:* information on HIV transmission, prevention and progression. Program content addressed strategies and skills building for resisting the social, cultural and interpersonal pressures to engage in sexual intercourse, sessions to combat stigmatization of people living with or affected by HIV and care of people with AIDS. *Activities:* teachers and peer supporters delivered classroom lessons, facilitate HIV and AIDS learning using anonymous question boxes, information corners, school health clubs and other school activities (assemblies and literary performance). *Dose/frequency/duration:* once per week over usual school period. *Theory used:* Social Learning Theory.	Control schools received the country’s ministry of education, science and technology guidelines for HIV/AIDS education, but had no PSABH trained teachers or Peer supporters in the schools.	18 months and 30 months
Mba 2007	Secondary schools, Nigeria	Randomised Controlled Trial	Age range: 10–20 Number: 360	*Objectives:* to improve knowledge of reproductive health and attitudes towards reproductive health issues. *Content:* information on STIs including HIV and family planning were provided during a workshop. *Activities:* a workshop on sexually transmitted diseases, HIV/AIDs, and family planning. *Dose/frequency/duration:* three hours. *Theory used:* NR	No intervention	6 weeks
Menna 2015	Secondary Schools, Ethiopia	Quasi-experimental	Age range: 15–18 (for about 80 % of the participants). Number: 560	*Objectives*: to prevent and control HIV/AIDs epidemic by changing knowledge, attitudes and practices of school youths in urban Ethiopia. *Content:* topics related to the structure and functions of human reproductive system, HIV/AIDS, prevention methods of HIV and risky sexual behaviours. *Activities*: peer educators were trained to educate peers on structure and function of reproductive organs, HIV/AIDs, risky sexual behaviours and methods of prevention of HIV. *Dose/frequency/duration:* at least 40 min, two sessions per week. *Theory used*: NR	No intervention	3 months
Michielsen 2012	Secondary schools, Rwanda	Non-randomised Controlled Trial	Mean age: 18.41 (intervention group) and 17.60 (control group). Number: 1950	*Objectives:* to reduce risky sexual behaviours and promotes sexual/productive health through anti-AIDs-clubs. *Content:* training of peers consisted of provision of information on HIV/AIDS, sexually transmitted diseases, family planning and pregnancies, the role of the peer educator and teaching methods including message transmission and counselling. *Activities:* peer educators teach students through group and individual counselling, songs, drama and other interactive activities to adopt positive and responsible behaviours. *Dose/frequency/duration:* NR *Theory used:* Theory of Reasoned Action, Social Learning Theory, Diffusion of Innovations Theory and Health Belief Model.	No intervention	6 months, 12 months
Okonofua 2003	Secondary schools, Nigeria	Randomised Controlled Trial	Mean age: 17.4 (intervention group) and 18.2 (control group). Number: 1247	*Objectives:* an intervention to improve STI treatment-seeking behaviour and reduce STI prevalence among Nigerian youths. *Content:* information on STIs and treatment were provided during health club activities. *Activities:* (1) establishment of reproductive health clubs in schools that organises campaigns during which health professional provide factual information on STI and treatment. Other activities include: (1) distribution of IEC materials, organizing debates, symposia, drama, essay writing, film show on STI treatment and prevention; (2) training of peer educators to provide counselling to peers as well as distribute IEC material on STI and refer those who have symptoms of STIs to health care providers; and (3) training of health care providers (medical practitioners, patent medicine dealers and Pharmacist) with emphasis on treatment algorithms, condom promotion and partner tracing with treatment. *Dose/frequency/duration:* 11 months. *Theory used:* NR	No intervention	10 months
Rijsdijk 2011	Secondary schools, Uganda	Quasi-experimental	Mean age: 16.1 Number: 1986	*Objectives:* To build self-esteem, personal decision making, self-identity, sexual development, role of social environment, gender equity, sexual/reproductive right and sexuality. *Content:* lessons focused on developing self-esteem, personal decision-making, gaining insights into a person’s identity and sexual development, the role of the social environment (e.g., peers, family, close friends, teachers, and media), gender equity, sexual and reproductive rights, sexuality issues, sexual health problems and the life skills necessary to know how to avoid or deal with them. *Activities:* low-tech, computer-based interactive sex education. Participants also develop IT and creative skills, which improve their job prospects. *Dose/frequency/duration:* 14 lessons over a period of six months. *Theory used:* Theory of Planned Behaviour and Health Belief Model.	The comparison received nothing while waiting to receive intervention (waiting-list control group).	Immediate
Ross 2007	Primary school and Health Centres, Tanzania	Community Randomised Trial	Age range: 14–≥ 18 Number: 13814	*Objectives:* to reduce the incidence of HIV, STI and unwanted pregnancy by providing knowledge and skills to enable youth reduce sexual risk, delay sexual debut and appropriate use of health services for sexual health issues. *Content:* topics covered included what is reproductive health and why is it important?; leaving childhood: Puberty; what are HIV and AIDS?; the facts about AIDS; the facts about sexually transmitted diseases; girls and boys have equal abilities; misconceptions about sex; refusing temptations; saying ‘No’ to sex; sexually transmitted diseases: Going to the clinic; how HIV infection causes AIDS; how Sexually Transmitted Diseases are spread; the relationship between HIV and sexually transmitted diseases; the reproductive organs and their functions; pregnancy and menstruation; respecting other people’s decisions; recognising and avoiding temptations; protecting yourselves: What are condoms?; how to avoid HIV infection and AIDS; Sexually Transmitted Diseases and their consequences; making good decisions; practising saying ‘No’; being faithful; achieving your future expectations; planning for your future; and protecting yourself: Correct use of condoms & the truth about condoms. *Activities:* (1) In–school interactive teacher led and peer led programme for primary school years 5–7. (2) Provision of youth friendly health services. (3) Distribution and promotion of condom use in the community. (4) Community mobilization activities including initial mobilization week and health weeks annually. Multiple activities were utilised across the four components of the intervention including question and answer, guided discussions, story reading, flip chart illustrations, role-plays and a scripted drama serial performed by class peer educators. it also includes: games; poems; comedy; video films; peer counselling; adult involvement; printed materials (pamphlets, brochures, manuals); awareness workshops for district council officials, religious leaders and ward development committee; condom distribution; and Youth Health Weeks held once a year, where interschool competitions take place *Dose/frequency/duration:* 12, 40-min sessions per year over 3 years. *Theory used:* Social Learning Theory.	No intervention	12 months, 36 months and 96 months (8 years)
Stanton 1998	Secondary schools, Namibia	Randomised Controlled Trial	Mean age: 17 Number: 515	*Objectives:* to improve basic knowledge on reproductive biology, HIV/AIDs, and risky behaviours. *Content:* the curriculum focused on improving knowledge of reproductive biology, risky behaviours (alcohol, substance abuse, and partner violence), HIV/AIDs, communication skills and framework for decision-making. *Activities:* variety of narratives, facts, games and exercises coupled with questions and discussions embedded in each session. *Dose/frequency/duration:* two-hour length per session (14 sessions) over 7 weeks. *Theory used:* Protective Motivational Theory.	Delay-control condition i.e., received intervention after the six month of follow up.	Immediate, 6 months and 12 months
Taylor 2014	High schools, South Africa	Randomised Controlled Trial	Mean age: 14.25 (intervention group) and 14.22 (control group). Number: 821	*Objectives:* to provide information that will improve attitudes and encourage intention to prevent teenage pregnancy. *Content:* topics include knowing yourself, the choice is yours, relationships, making choices, body development, contraception, peer pressure, culture, parenthood, responsibility, and human rights and gender norms. *Activities:* role-play, debates, small and large group discussion, and videos viewing to start up discussions with students. *Dose/frequency/duration:* 12 weekly. *Theory used:* I-change model.	School life skills programmes.	4 months and 8 months
Tibbits 2011	High schools, South Africa	Randomised Controlled Trial	Mean age: 14.0 Number: 4040 (second cohorts) 2383 (first cohorts)	*Objectives:* to increase knowledge, promote social, emotional and refusal skills on substance use and sexual behaviours as well as encouraging the use of healthy free time. *Content:* topics include social-emotional skill programmes such as decision-making and self-awareness and positive use of time like beating boredom, and leisure motivations. Specific lessons on attitudes, knowledge, skills surrounding sexual risk and substance use were also included. *Activities:* teachers delivered class lessons. *Dose/frequency/duration:* 12 lessons and 6 booster lessons (each lesson 2–3 class periods). *Theory used:* Self-Determination Theory, Selective Optimization with Compensation and Social Cognitive Theory.	Students in the comparison schools received the government mandated Life Orientation curriculum, which differ substantially between schools and overlap minimally with HealthWise content.	12 months, 18 months and 24 months
Van der Maas 2009	Secondary schools, Nigeria	Quasi-experimental	Age range: 10–30 Number: 250	*Objectives:* to increase HIV/AIDS awareness and HIV life skills. *Content:* teaching included relevant topics on HIV and life skills. *Activities:* sketches, songs, rallies, competitions and videos with scenarios from Africa translated into the local language. UNPFA/UNAIDS peer education toolkit and Family Health International peer-to-peer training guide manuals were used. *Dose/frequency/duration:* NR *Theory Used:* NR	The control group did not receive any peer education.	24 months
Ybarra 2013	Secondary schools, Uganda	Randomised Controlled Trial	Age range: 13–19 Number: 366	*Objectives:* to provide information about HIV, decision making and communication, motivations to be healthy, proper use of condom and healthy relationships. *Content:* modules were on information about HIV including prevention; decision-making and communication; motivations to be healthy; how to use a condom to be healthy; and healthy relationships. *Activities:* self-administered computer interactive sessions. *Dose/duration/frequency:* One hour per module (six modules) over six weeks. *Theory used*: Information-Motivation-Behaviour model.	The control arm was ‘treatment as usual’: Participants in the control arm received no programming or interaction beyond the HIV programming that was currently being offered at their school as part of their usual schedule of extracurricular activities.	3 months and 6 months

### Methodological quality of included studies

**Fig. 2:**
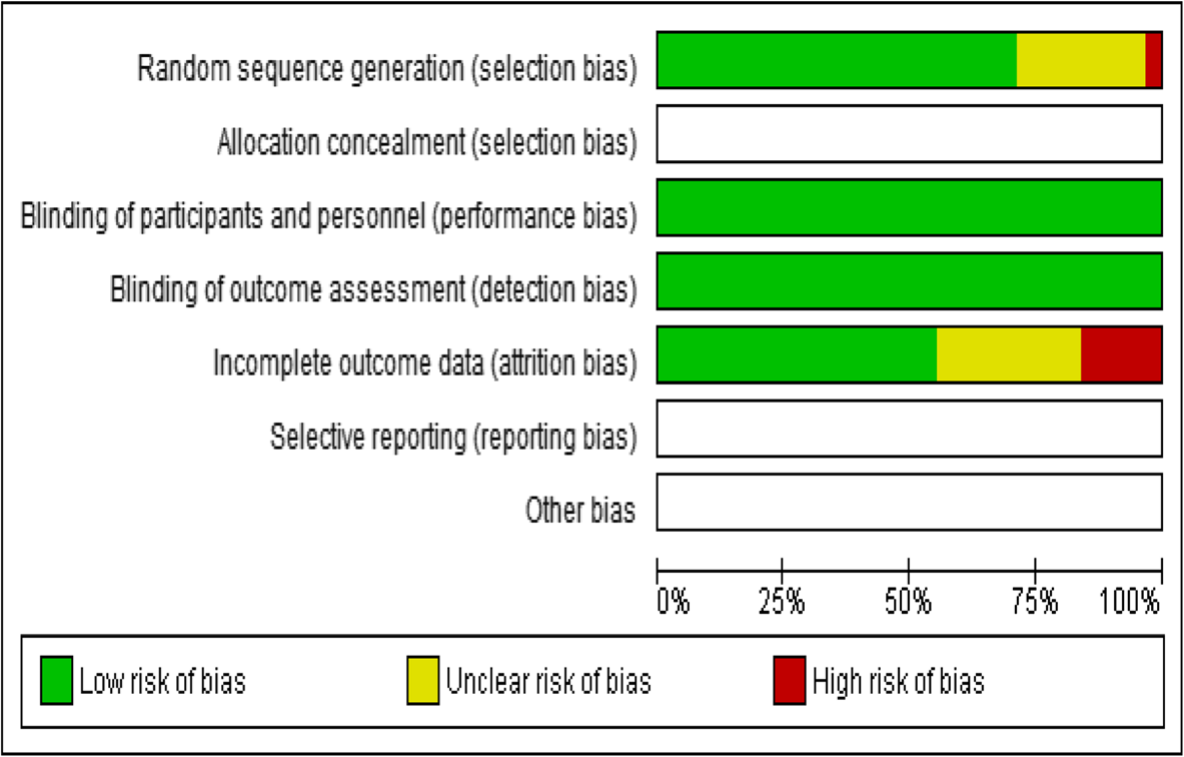
Risk of bias graph

**Fig. 3:**
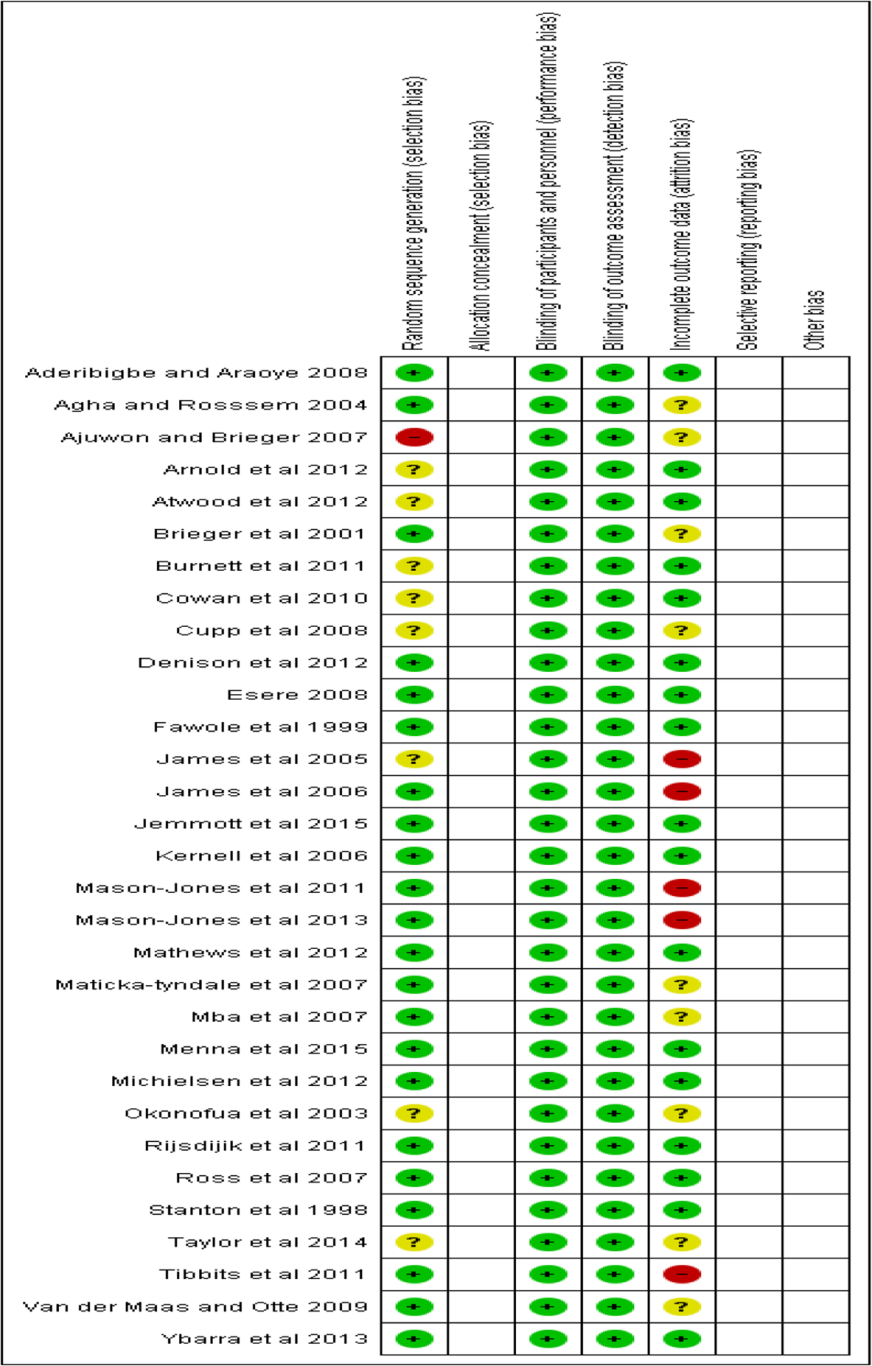
Risk of bias summary

Methodological quality was generally high; 20 of the included studies were assessed as having “low”, 8 as “moderate” and 3 “high” risk of bias (see Additional file 5: Table S5, which contains the quality assessment process). Two of the high risks of bias studies [43, 65] were found to be at risk of attrition bias due to loss to follow up of more than 30 % and ‘unclear’ selection bias. The other high-risk study [66] was assessed to be at risk of selection bias because the baseline characteristics of confounders differed between the two arms of the intervention, which were not adjusted for in the analysis, and ‘unclear’ attrition bias. See Figs. 2 and 3 for risk of bias graph and risk of bias summary for each study respectively.

### Description of interventions

All included interventions delivered comprehensive sexual health education in classroom settings (see Table 1 for interventions description). Comprehensive SBSHE provides participants with information on transmission of sexual infections, safer sex practices and prevention of STIs and unwanted pregnancies [67], in contrast to abstinence-only interventions. Various intervention delivery methods were employed in one or more combinations some of which include lectures or presentations (*n* = 6), group discussions (*n* = 14), role-plays or dramas (*n* = 14), and distribution of information, education and communication (IEC) materials (*n* = 6). Films shows or audio vignettes (*n* = 7), songs (*n* = 4), counselling (*n* = 6), quiz and essay competitions (*n* = 7) were also used. Condoms were distributed in three interventions [35, 62, 66]. Two were interactive computer-based programmes [68, 69] and one only involved provision of a printed material [65].

The dose and duration of the interventions varied widely and ranged from a single 1 h and 45 min [70] session to multiple sessions delivered over 36 months [35]. However, in general, the interventions employed one session per week of 30–60 min duration over a period of 6–12 weeks [64, 68, 71–76]. Fourteen theories were said to inform the design of 16 of the interventions with Social Learning Theory (*n* = 6), Social Cognitive Theory (*n* = 3) and Theory of Planned Behaviour (*n* = 3) being most frequently used.

### Implementation details

Seven of the interventions were delivered by both teachers and peer educators [35, 41, 51, 66, 69, 73, 75]; similarly, seven by peer educators [62, 70, 77–81]; and three by teachers only [47, 72, 82]. Health educators, community physicians, volunteer adults or youths, nurses or other health personnel were involved in delivering six of the interventions [43, 58, 63, 71, 74, 83] and one was delivered by the researchers that developed it [84]. Twenty-one of the 31 interventions reported that the facilitators received some form of training (see Additional file 6: Table S6, which contains implementation details of the included interventions).

Only twelve (of 31) studies reported monitoring of implementation and only seven of these [35, 47, 58, 68, 69, 75, 82] reported on fidelity of implementation. Just two studies [35, 75] reported that fidelity had been achieved and explained how fidelity had been assessed. In other cases lack of compatibility with local circumstances undermined fidelity of delivery. For example, in the intervention evaluated by Mathews et al. [47] some teachers did not implement condom demonstrations and other skilled-based activities due to overwhelming large number of students per class. Similarly, in the intervention evaluated by Rijsdijk et al. [69] poor availability of computers meant that the intervention had to be modified to delivery through print materials. Sub-sample analyses in this evaluation showed that schools with ‘complete’ implementation had most of the significant positive effects compared to those with ‘partial’ implementation [69]. Complete implementation schools are those where the teachers fully implemented more than 50 % of the 14 lessons in the programme.

### Outcomes

Three studies reported STI outcomes [35, 55, 58]. Two [35, 58], measured HIV infections close to the median follow-up period of 54 months. Cowan et al. [58] found no evidence of an effect on HIV infections among males or females (adjusted odds ratio (*aOR)* = 1.20, 95 % CI = 0.66–2.18 and *aOR* = 1.15, 95 % CI = 0.81–1.64 respectively). Ross et al. [35] reported incidence rate per 1000 person-years and the intervention also did not significantly reduce HIV infection risk for both short (adjusted rate ratio (aRR) = 0.75, CI = 0.34–1.66 for young women) and long-term follow-up periods (adjusted prevalence rate (*aPR*) = 0.91, *CI* = 0.50–1.65 for men and *aPR* = 1.07, *CI* = 0.68–1.67 for women).

**Fig. 4:**
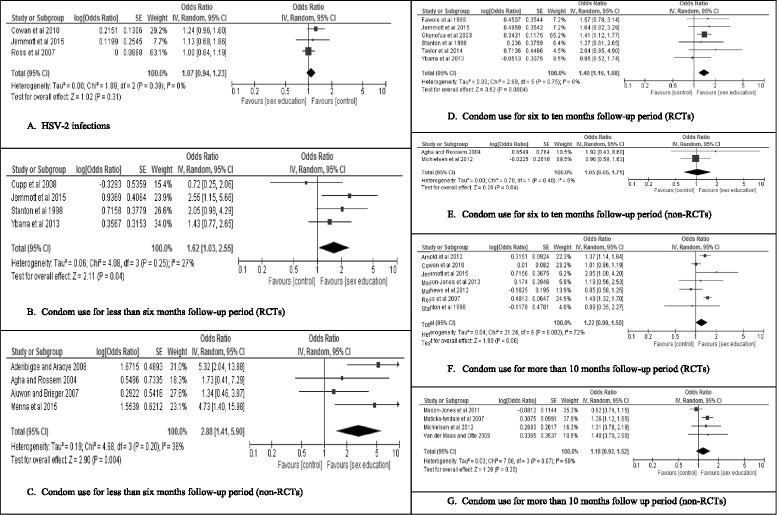
Forest plots for meta-analysis. **a** HSV-2 infections. **b** Condom use for less than six months follow-up period (RCTs). **c** Condom use for less than six months follow-up period (non-RCTs). **d** Condom use for six to ten months follow-up period (RCTs). **e** Condom use for six to ten months follow-up period (non-RCTs). **f** Condom use for more than 10 months follow-up period (RCTs). **g** Condom use for more than 10 months follow up period (non-RCTs)

Three studies [35, 55, 58], also measured HSV2 infections for median follow-up period of 54 months. SBSHE showed no statistically significant effect in reducing the risk of this infection (*OR* = 1.07, 95 % *CI* = 0.94–1.23, *p* = 0.31) (Fig. 4 Panel a). Ross et al. [35] also did not find any significant effect at long-term follow-up (*aPR* = 0.94, *CI* = 0.77–1.15 for males and *aPR* = 0.96, *CI* = 0.87–1.06 for females) and similarly, the intervention by Jemmott III et al. [55] did not find a significant effect at 42-month follow-up period.

Ross et al. [35] measured other STIs including Syphilis, Chlamydia, Gonorrhoea and Trichomonas and found no statistically significant difference between the intervention and control group in the prevalence of these infections for both short and long-term follow-up periods. However, the intervention by Jemmott III et al. [55] significantly reduced curable STIs (Chlamydia, Gonorrhoea and Trichomonas) at 42-month follow-up period (*OR* = 0.71, 95 % *CI* = 0.54–0.95), but not at 54-months follow-up (*OR* = 1.15, 95 % *CI* = 0.84–1.57).

All 31 studies assessed self-reported condom use. Fifteen of the interventions [35, 41, 43, 51, 55, 62, 64, 71, 72, 75, 77, 79, 82, 83, 85] resulted in statistically-significant increases in condom use while 16 showed no statistically-significant increases in condom use [47, 58, 63, 65, 66, 68–70, 73, 74, 76, 78, 80, 81, 84, 86]. No intervention resulted in statistically significant reductions in self-reported condom use.

Twenty of the studies that measured condom use provided adequate data to enable inclusion in meta-analyses [35, 41, 47, 51, 55, 58, 63, 66, 68, 70, 73, 74, 76, 77, 79–81, 83, 85, 86]. Measures of condom use at last sex [35, 41, 47, 51, 58, 63, 66, 70, 73, 74, 79–81, 83, 85, 86], consistent condom use in the last 12 months [77], condom use [76], condom use in the past three months [55] and 100 % condom use in the last three months [68] were in the meta-analysis. All of these measures were coded by the original authors as dichotomous use/non-use scores. For short-term follow-up of less than 6 months, intervention participants were more likely to report condom use in both RCTs (*OR* = 1.62, 95 % *CI* = 1.03–2.55, *p* = 0.04) (Fig. 4 Panel b) and non-RCTs (*OR* = 2.88, 95 % *CI* = 1.41–5.90, *p* = 0.004) (Fig. 4 Panel c). Similarly, intervention participants were more likely to use condoms at intermediate follow-up of 6–10 months with the effect being statistically significant for the RCTs (*OR* = 1.40, 95 % *CI* = 1.16–1.68, *p* = 0.0004) (Fig. 4 Panel d) but not for non-RCTs (*OR* = 1.05, 95 % *CI* = 0.65–1.71), *p* = 0.84) (Fig. 4 Panel e). At follow-up of more than 10 months, the effect was marginally significant for RCTs (*OR* = 1.22, 95 % *CI* = 0.99–1.50, *p* = 0.06) (Fig. 4 Panel f) and non-significant for non-RCTs (*OR* = 1.18, 95 % *CI* = 0.92–1.52, *p* = 0.20) (Fig. 4 Panel g).

Sub group analyses showed no effect of type of condom use measure on condom use but greater condom use effects when crude, compared to adjusted ORs, were employed (see Additional file 7: Table S7, Panel A-H).

All authors were emailed to acquire missing data. Nonetheless, three studies [43, 71, 82] were excluded from the meta-analysis because they reported only *ORs* without *CI*, standard error of mean or *p*-value, precluding further analysis. Another study [78] was also removed because the follow-up period was unclear. Another seven studies [62, 64, 65, 69, 72, 75, 84] measured condom use using continuous measures composed of differing items and could not be included. James et al. [82] measured consistent use of condom in the preceding 6 months by assessing whether a condom was used during all intercourse instances, sometimes or not at all. Mba et al. [84] assessed whether participants practised or intent to practise condom use, as a STI-prevention technique. Esere [64] used a 4-point Likert At-Risk Sexual Behaviour Scale which includes ‘do not use condoms while having sex’ as a component of the scale. The Ugandan study ‘The World Starts with Me’ used a 4-item condom use performance behaviour scale to measure condom use [69] and Burnett et al. [72] measured condom use using a 14-item scale. Frequency of condom use was measured on a scale of one (never) to six (always) in the study by Karnell et al. [75]. Finally, Brieger et al. [62] measured contraceptive information including condom use, pills and foaming tablets. This variability of outcome measures prevented the inclusion of these studies in our meta-analysis. Seven of the studies [43, 62, 64, 71, 72, 75, 82] not in the meta-analysis found statistically significant results in increased condom use in the intervention group compared to the control group (see Table 2 which contains results and scales used to measure condom use for studies not included in the meta-analysis).

**Table 2:** Studies That Reported Condom Use Not Included In Meta-Analysis

Intervention	Scale used to measure condom use	Finding
At wood et al. 2012	Use/non-use score	Significant effect of increased consistency of condom use in the last three months at 9-month follow-up period for sexually active participants at baseline and controlling for baseline condom use (*B*_*9mth*_ = 0.032, *p* < 0.05).
Burnett et al. 2011	14-item scale	Statistically significant difference in positive direction between the intervention and control group of the study (*F* = 32.39, *p* < 0.001).
Brieger et al. 2001	Measured modern contraceptive use including condom use, pills and foaming tablets.	Found significantly increased reported modern contraceptive use in the intervention group compared to the control (Fisher’s exact *p* = 0.004).
Denison et al. 2012	Use/non-use score	No evidence of difference in reported condom between the intervention and control group at follow up (*aOR* = 0.93, 95 % *CI* = 0.57–1.53)
Esere 2008	A 4-point Likert At-Risk Sexual Behaviour Scale which include ‘do not use condoms while having sex’ as a component of the scale.	Significant difference between the intervention and control group (*F* = 95.93, *p* < 0.05).
James et al. 2005	Use/non-use score	The intervention (reading Laduma once) was found to have no significant effect on Consistent condom use six weeks post intervention.
James et al. 2006	Consistent use of condom in the preceding six months was measured by assessing whether condom was used all the time, sometimes or not at all.	Full implementation group used condom more at last sex (*B* = −0.80, *SE* = 0.40, Wald (1, 57) = 4.16, *p* < 0.05, *OR* = 0.45). However, no effect was found for partial implementation group compared with the full implementation (*B* = −0.21, *SE* = 0.41, Wald (1,157) = 0.27, *p* = 0.60, *OR* = 0.81)
Karnell et al. 2006	Measured frequency of condom use on a scale of 1 (never) to 6 (always).	Participants in the intervention group have significantly higher scores than those in the control group (*p* < 0.05).
Mba et al. 2007	Practised or intended to practised STI prevention technique (specifically condom use).	All sexual active participants in the intervention group practised or intended to practise STI prevention technique at six-week follow up compared to 18 participants at baseline. However, in the control group no change before and after the intervention.
Rijsdijk et al. 2011	Condom use measured with four-item condom use behaviour (e.g., How often have you obtained a condom in the past 6 months” and “in the past 6 months, did you use a condom when having sex” α = 0.84)	No significant effects of the intervention was found in ‘past performance behaviour’ including condom use (*F* = 0.46).
Tibbits et al. 2011	Use/non-use score	Significant effect in the positive direction (*β* = −0.16, *SE* = 0.08, *p* < 0.05) for risk at last sex (including condom use with partner at last sex) among virgins at baseline. However, non-significant effect was found for all participants (virgins and non-virgins at baseline). Similarly, non-significant effect was found for condom less sex refusal outcome for all participants including virgins at baseline. Similarly, no difference observed in proportion of participants that reported sexual intercourse in consistent condom use at wave 4 and wave 5 of the study.

### Quality of evidence and summary of findings

Table 3 shows the summary of findings and quality of evidence for outcomes included in meta-analyses. The quality of evidence for HSV-2 infection, condom use for 6–10 months and more than 10 months follow-up among RCTs is ‘high’, which means we are very confident that the true effect lies close to the estimate. We are moderately confident in the evidence for self-reported condom use for less than 6 months follow-up among the RCTs. For the remaining outcome categories, we have limited to very low confidence in the proximity of the estimates to the true effects.

**Table 3:** Quality of evidence and summary of findings table

	Quality assessment							№ of participants		Effect		Quality
Outcome (follow up period)	№ of studies	Study design	Risk of bias^a^	Inconsistency^b^	Indirectness^c^	Imprecision	Publication or reporting bias	School-based sexual health education	Nothing or usual curriculum	Relative (95 % CI)	Absolute (95 % CI)	
Herpes Simplex Virus- 2 (54 months)	3	randomised trials	not serious	not serious	not serious	not serious^d^	none	746/6146 (12.1 %)	738/6127 (12.0 %)	OR 1.07 (0.94 to 1.23)	7 more per 1,000 (from 6 fewer to 24 more)	⨁⨁⨁⨁ HIGH
*Condom use (less than six months)	4	randomised trials	not serious	not serious	not serious	serious^e^	none	332/741 (44.8 %)	295/720 (41.0 %)	OR 1.62 (1.03 to 2.55)	120 more per 1,000 (from 7 more to 229 more)	⨁⨁⨁◯ MODERATE
*Condom use (less than six months)	4	quasi-experimental trials	not serious	not serious	not serious	Serious^f^	None	90/226 (39.8 %)	63/197 (32.0 %)	OR 2.88 (1.41 to 5.90)	255 more per 1,000 (from 79 more to 415 more)	⨁⨁◯◯ LOW
*Condom use (six to 10 months)	6	randomised trials	not serious	not serious	not serious	not serious^g^	none	485/1238 (39.2 %)	494/1494 (33.1 %)	OR 1.40 (1.16 to 1.68)	78 more per 1,000 (from 34 more to 123 more)	⨁⨁⨁⨁ HIGH
Condom use (six to 10 months	2	quasi-experimental trials	not serious	not serious	not serious	serious^h^	publication bias strongly suspected^i^	62/146 (42.5 %)	55/131 (42.0 %)	OR 1.05 (0.65 to 1.71)	12 more per 1,000 (from 100 fewer to 133 more)	⨁◯◯◯ VERY LOW
*Condom use (more than 10 months)	7	randomised trials	not serious	not serious	not serious	not serious^j^	none	2955/8106 (36.5 %)	2678/8868 (30.2 %)	OR 1.22 (0.99 to 1.50)	43 more per 1,000 (from 2 fewer to 92 more)	⨁⨁⨁⨁ HIGH
Condom use (more than 10 months)	4	quasi-experimental trials	not serious	not serious	not serious	Serious^k^	none	1442/2205 (65.4 %)	1647/2649 (62.2 %)	OR 1.18 (0.92 to 1.52)	38 more per 1,000 (from 20 fewer to 92 more)	⨁◯◯◯ VERY LOW

*CI* Confidence interval, *OR* Odds ratio, *outcomes with statistically significant positive results

^a^Quality was assessed as ‘not serious’ for all outcomes because majority of the studies included were of ‘low’ to ‘moderate’ risk of bias

^b^I^2^ were all below 75 % and therefore, quality was assessed as ‘not serious’ for all the outcomes

^c^Quality was assessed as ‘not serious’ for all outcomes because the interventions were fairly similar, participants were adolescents or young adults and all assessed similar outcomes (condom use and laboratory test of Herpes Simplex Virus-2)

^d^Relative risk increase or decrease is less than 25 % (95 % CI of 0.97−1.23)

^e^Relative risk increase or decrease is greater than 25 % (95 % CI of 1.03 to 2.55)

^f^Relative risk increase or decrease is greater than 25 % (95 % CI of 1.41 to 5.90)

^g^Relative risk increase or decrease is very close to 25 % confidence interval (95 % CI of 1.16 to 1.68)

^h^Relative risk increase or decrease is greater than 25 % (95 % CI of 0.65 to 1.75)

^i^Funnel plot was asymmetrical

^j^Relative risk increase or decrease is very close to 25 % (95 % CI of 0.99 to 1.50)

^k^Relative risk increase or decrease is greater than 25 % (95 % CI of 0.92 to 1.52)

### Features of effective interventions

Small study samples mean that interpretation of the distribution of characteristics across interventions that did or did not result in increased condom use can only be tentative (see Table 4). Nonetheless, we can observe that effective interventions were more often adapted from other programmes, were theory-based, included provision of health services, included activities outside school and were implemented with fidelity.

**Table 4:** Frequencies of occurrence of features associated with effectiveness

Intervention Characteristic	Interventions with benefit (*N* = 15)	Interventions without benefit (*N* = 16)
Need assessment of target participants and involvement of stakeholders (parents, teachers or students) in designing the intervention	9 (60 %)	8 (50 %)
Adapting from other programs or curriculum that are found to be efficacious.	7 (47 %)	2 (13 %)
Theory-based	9 (60 %)	5 (29 %)
Skilled-based	10 (67 %)	9 (56 %)
Provision of adolescents health services	3 (20 %)	1 (6 %)
Distribution of condoms	2 (13 %)	1 (6 %)
Activities outside school environment	6 (40 %)	2 (12 %)
Training of facilitators	10(67 %)	11 (65 %)
Implementation of intervention with fidelity	2 (13 %)	0 (0.00)

*N* number of studies

## Discussion

We conducted a comprehensive review of school-based sexual health education interventions in sub-Saharan Africa evaluated using experimental or quasi-experimental methods. Given the need for public health interventions to reduce sexually transmitted infections, including HIV, and the potential effectiveness of school-based sexual health interventions, the most striking finding is paucity of published evaluations. Across 31 interventions meeting our inclusion criteria, we found no evidence of effectiveness in reducing STIs, including HIV, although one study [55] reported a reduction in curable STIs (Chlamydia, Gonorrhoea and Trichomonas) at one follow-up period. We also found no evidence of harm. This mirrors the findings of previous, comprehensive reviews [22, 25, 87, 88]. More rigorous evaluations of best practice, sustainable, school-based sexual health programmes in sub-Saharan Africa are needed.

Interventions were effective in increasing self-reported condom use and, encouragingly, the positive effect on condom use was stronger among evaluations employing more robust experimental methods (RCTs) for intermediate and long-term follow-up periods. Previous reviews [9, 11, 26] have also found methodically stronger studies to be associated with stronger effects. Perhaps, unsurprisingly, short and intermediate (versus, long follow-up periods) yielded greater condom use gains, suggesting that further intervention may be needed to sustain behaviour change [11]. These findings contrast with previous suggestions that SBSHE in sSA has a poor record of changing sexual behaviours including condom use [8–10] and supports further investment in SBSHE to promote condom use in low-and middle-income countries [5, 25].

Tentative consideration of characteristics found in interventions that did or did not result in statistically-significant increases in condom use recommends that intervention designers should consider adapting interventions from pre-existing effective programmes, base their interventions on theory-based logic models of mechanism and link them to health service provision including condom distribution. Finally, intervention designers need to ensure that they assess fidelity and take steps to ensure that interventions are delivered as designed. In this review, just two studies [35, 75] reported on fidelity of delivery. It was impossible, therefore, to determine whether or not the interventions were delivered as intended and whether this determined effectiveness. This review highlights the need for further rigorous evaluations of SBSHE to assess impact on incidence or prevalence of STIs including HIV. In addition, future evaluations need to assess and report on implementation processes including fidelity. This will provide better guidance on how and why interventions ‘work’ or ‘do not work’.

Limitations in the available data recommend caution in interpretation of our findings. For example, condom use reporting is subject to social desirability bias and recall bias, although guidance is available on measures that may minimise such bias [89]. Greater consistency in use of best measures of condom use would assist data synthesis, although sensitivity analysis did not reveal differences in effectiveness as a result of the self-report measures used. Ideally, a larger sample of studies would have been available and further moderators of effectiveness could have been considered. In particular, we would have liked to report on whether interventions in primary or secondary schools were more or less effective and whether studies with greater or lesser risk of bias tended to result in greater increases in condom use. However, for both these sub-group analyses multiple cells included just one study across follow up points. The four evaluations of primary school interventions [35, 41, 55, 71] suggest that these can be just as effective as secondary school interventions and this may indicate that early school-based intervention is likely to be more effective. Similarly, we would have liked to assess whether effects were greater or lesser for young men and women but only four studies [35, 41, 58, 79] included in the meta-analysis presented separate gender analyses. It is worth noting too that because blinding is impossible in relation to school-based sexual health education, we were only able to employ two critical, of four dimensions of the Cochrane Collaboration Tool assessed to score the overall risk of bias of the studies in this review. Reviewing studies published in English may have limited our sample. In addition, although double screening of 500 randomly selected title and abstract entries showed near perfect agreement between two reviewers (generating an ACI score of 0.98) further double screening at this stage could have been conducted.

## Conclusion

We conducted a review of school-based sexual health education interventions in sub-Saharan Africa. Interventions to safeguard adolescents from sexually transmitted infections, including HIV are especially needed in sub-Saharan Africa. School-based interventions have the potential to be inclusive and to provide comprehensive preventive education and training. We assessed the impact of such interventions on incidence or prevalence of STIs and self-reported condom use. We also identified characteristics associated with effective interventions. We found no effect of the interventions on STIs, however, some positive effect on condom use was observed. Certain features present among interventions effective in promoting condom use were observed. Despite limitations, our review indicates that school-based sexual health education may be an effective strategy to promote condom use among sub-Saharan African adolescents over periods of up to 10 months. Interventions may be optimised by including features found in previous effective programmes. Above all, this review highlights the need for further rigorous evaluations of school-based sexual health education interventions in sub-Saharan Africa including assessment of infection prevalence and fidelity of delivery. Guidance on reporting implementation processes including fidelity would be helpful to intervention designers.

## Additional files

Additional file 1: Review Protocol. (DOCX 71 kb)
Additional file 2: Search Strategy for Medline Which Was Modified and Used In Other Databases. (DOCX 21 kb)
Additional file 3: Data Extraction Form. (DOCX 26 kb)
Additional file 4: Lists of Excluded Studies with Reasons for the Exclusion. (DOCX 28 kb)
Additional file 5: Modified Cochrane Collaboration Tool for Assessing Risk of Bias. (DOCX 45 kb)
Additional file 6: Implementation Details. (DOCX 33 kb)
Additional file 7: Forest Plots for Sensitivity Analyses. (DOCX 96 kb)

## Abbreviations

AIDS: Acquired immune deficiency syndrome
aOR: Adjusted odds ratio
aPR: Adjusted prevalence rate
aRR: Adjusted rate ratio
CI: Confidence interval
GRADE: Grading of recommendations assessment, development and evaluation
HIV: Human immunodeficiency virus
HP4RY: HIV prevention intervention for rural youth
HSV2: Herpes simplex virus-2
*I*^*2*^: I-squared statistic
IEC: Information, education and communication
MkV: *Mema Kwa Vijana*
NR: Not reported
OR: Odds ratio
PSABH: Primary school action for better health
RCT: Randomised controlled trial
SATZ: Promoting sexual and reproductive health, school-based HIV/AIDS intervention in sub-Saharan Africa
SBSHE: School-based Sexual Health Education
SoF: Summary of findings table
sSA: Sub-Saharan Africa
STI: Sexually transmitted infection
