# Supplementary material for: School-based sexual health education interventions to prevent STI/HIV in sub-Saharan Africa: a systematic review and meta-analysis
Source: BMC Public Health. 2016 Oct 10;16:1069. doi: 10.1186/s12889-016-3715-4 (PMC5057258; doi:10.1186/s12889-016-3715-4)
Supplement: Additional file 1: — Review Protocol. (DOCX 71 kb) [file 12889_2016_3715_MOESM1_ESM.docx]

**SUPPLEMENTARY FILE 1: REVIEW PROTOCOL**

**SCHOOL-BASED SEXUAL HEALH EDUCATION INTERVENTIONS FOR PREVENTION OF SEXUALLY TRANSMITTED INFECTIONS IN SUB-SAHARAN AFRICA**

**BACKGROUND**

Over one million cases of sexually transmitted infections (STIs) occur daily worldwide, with approximately 500 million people living with curable STIs including Chlamydia, Gonorrhoea, Syphilis and Trichomoniasis (WHO, 2013). STIs are the most common cause of health care visits and in developing countries, it causes significant loss of productivity to individuals and communities due to high prevalence (CDC, 2008). The burden is more in Africa where around 70% of world infections occur (Albini et al, 2013). Adolescents and young adults are among the high risk group with about half of new Human Immune Deficiency Virus (HIV) infections occurring in individuals aged 15-24 (Fonner, Armstrong, Kennedy, O’Reilly, & Sweat, 2014), more than 90% of which are acquired through sexual transmission. This is partly because adolescents are becoming sexually active at younger age worldwide with rapid change of partner which enhances the spread of STIs (Schaalma, Ph, Abraham, Gillmore, & Kok, 2004).

School-based sexual health education (SBSHE) is arguably the most comprehensive, uniform, universal and effective way of promoting sexual health among adolescent and young adults (Schaalma et al., 2004). It is also viewed as a necessary step in halting the spread of STIs to the general population (Gallant & Maticka-Tyndale, 2004). The interventions aim to increase knowledge and awareness about STIs which subsequently increase healthier behaviours like self-efficacy, consistent use of condom and delay onset of sexual activity (Burazeri, Roshi, & Tavanxhi, 2004; Lindberg & Maddow-Zimet, 2012; Wellings et al., 2006). Several studies have reported efficacy of SBSHE in reducing risky sexual behaviours among adolescents and young adults. Pual-Ebhohimhen et al (2008) found such interventions to increase knowledge but with least effect on actual behaviour change in sub-Saharan Africa. However, Napierala Mavedzenge et al (2010) found a clear reported evidence of decrease in risky sexual behaviours. Comprehensive SBSHE interventions with community component were found to be more effective than abstinence-only interventions in developing countries (Fonner et al, 2014).

Previous reviews of interventions to prevent STI/HIV among adolescents and young adults have been conducted in sub-Saharan Africa. While some of these reviews were not focused on school-based interventions or are restricted to HIV prevention only (Michielsen et al., 2010; Michielsen, Chersich, Temmerman, Dooms, & Van Rossem, 2012; Napierala Mavedzenge, Doyle, & Ross, 2011; & Wamoyi et al., 2014) , others are quite out of date (Medley et al, 2009; Ross et al, 2006; Paul-Ebhohimhen, Poobalan, & van Teijlingen, 2008). The reviews by Fonner et al (2014) and Amaugo et al (2014) are very recent and include only school-based interventions. However, the former include other developing countries outside sub-Saharan Africa and the latter focused only on one country (Nigeria). None of the reviews on SBSHE looked at the implementation factors that led to success or failure of the included interventions including fidelity of implementation.

There is clearly need for a review because Fonner’s et al (2014) review ‘missed’ some potentially eligible articles which are in Amaugo’s et al (2014) despite the fact that they were published within the same search year. Therefore, this makes the comprehensiveness and hence the generalizability of the findings questionable. In addition, the review by Paul-Ebhohimhen et al (2008) is somewhat out-dated. This review will be comprehensive and update previous reviews. It would also look at the features that are associated with effective interventions and implementation processes including fidelity.

This review aim to answer the following questions:

1. How effective are school-based sexual health education interventions in sub-Saharan Africa in promoting condom use and preventing sexually transmitted infections?
2. What characterizes effective school-based sexual health education interventions implemented in sub-Saharan Africa?
3. Are school-based sexual health education interventions implemented with fidelity in sub-Saharan Africa?

**CRITERIA FOR CONSIDERING STUDIES FOR THIS REVIEW**

***Study design***: Randomised controlled trials including cluster-randomised trials (schools or classrooms) and crossover trials will be included. Quasi-experimental designs will also be considered. The comparison can be usual intervention or nothing. Systematic reviews and review of reviews will be used to identify other trials. Other study designs will be excluded because of their potential for bias, which may lead to misleading evidence (Cochrane, 2014).

***Participants***: Children, adolescents and young adults in sub-Saharan Africa between the ages of 4 and 24 without restriction to any sex, ethnicity or nationality; that range falls within World Health Organization (WHO) (2014) definition of young people. Interventions whose majority (80%) of the participants fall within this age range will also be included.

***Interventions***: Interventions that were delivered in school setting or had a component delivered in school aiming at preventing sexually transmitted infections including HIV and/or unwanted pregnancy will be included. The intervention can be peer or adult led and may use multifaceted approach involving family or community. Authors may be contacted for studies that met the inclusion criteria but do not report the implementation process or components of interventions.

***Setting:*** Only studies that reported interventions that were conducted in sub-Saharan African primary or secondary schools will be included.

***Outcomes***: Studies will be included if STIs including HIV and/or condom use were the outcomes reported.

**SEARCH STRATEGY**

Electronic databases including Medline, PsycInfo, EMBASE, CINAHL, Web of Knowledge, The Cochrane Library, British Education Index/EBSCOhost, Australian Education Index/ProQuest, Education Research Complete/EBSCOhost and ERIC/ProQuest will be searched. Reference list of all included studies and identified similar reviews will be searched for additional studies that may be eligible. Google scholar and Science citation index will be used to identify more relevant citations from included studies. Contact with experts in the field will be made to ensure that all grey and unpublished studies are not missed. Hand searching of table of contents of relevant journals like Journal of Adolescent Health, Journal of Youth and Adolescence, AIDs and Behaviour, AIDS, AIDS Education and care, and AIDS care will be performed.

Search strategy will be developed using the population, intervention, outcome and design framework. See appendix for a draft of the search strategy for Medline, which was adapted from previous similar studies (Pienaar et al, 2011; & Amaugo et al, 2014) and modified. This will also be modified for use in other databases. To include as much articles as possible, no restriction on date of publication, however, only articles published in English will be included.

**STUDY SELECTION**

The first reviewer will screen the title and abstract of the search results, and a second reviewer will check a randomly selected percentage. Full text of potential studies that met the inclusion criteria will be obtained. The first reviewer will assess full text of potentially eligible articles for inclusion and second reviewer will check another randomly selected percentage. A second reviewer will check all studies that met the inclusion criteria. *AC1* statistics introduced by Gwet (2002) and argued to be the most valuable and reliable tool for determining the extent of inter-rater agreement will be used to assess the inter-rater reliability. Any controversy between the two reviewers will be resolved by discussion or seeking opinion of a third reviewer. Those that are excluded at this stage will be provided in a table of excluded studies together with reason(s) for exclusion. Attempt will be made to contact authors of potentially eligible studies that are unclear for clarification.

**STUDY QUALITY**

The Cochrane Tool of Bias will be used to assess the quality of included trials which is a domain-based evaluation that give critical assessment of each domain that bias may arise (Cochrane, 2011). This tool has the advantage of encouraging user to tailor to given scenario and adds to transparency in addition to emphasis on conduct rather that report, unlike most scales and checklists. Each domain is judged as ‘low’ ‘moderate’ and ‘high’ of bias using the Cochrane tool.

**DATA MANAGEMENT**

Endnote reference manager will be used to sort, remove duplicates and store studies retrieved from the databases. This will make management of large number of articles easier and thus reduce mistakes.

**DATA EXTRACTION**

Data will be extracted using a developed data extraction form, which will cover items that will help in answering the review aims. The first reviewer will extract data, which will be checked by a second reviewer to ensure accuracy. Data on the following will be extracted from the included studies: i) Author(s); ii) Setting; iii) characteristics of participants; iv) Detail of the intervention; v) implementation process of the intervention; vi) Duration and frequency of the intervention; vii) effectiveness of the intervention on outcome measures and period of follow-up; viii) makers of methodological quality; and ix) features of effectiveness. Attempts will be made to contact authors for missing or incomplete data.

**ASSESSMENT OF HETEROGENEITY**

Random effect method instead of fixed effect method will be used to weight the studies if clinical and methodological diversity is found to be wide (Cochrane, 2011). Statistical heterogeneity will be tested using Chi^2^ test with P < 0.1 and I^2^ value of 25%, 50% and 75% as low, moderate and high heterogeneity respectively (Higgins et al, 2003). If high heterogeneity is found, trials will not be statistically combined. Instead, attempts will be made to find possible clinical or methodological reasons for this variation.

**DATA SYNTHESIS**

Meta-analysis may be performed with studies that report adequate information to enable the analysis. These analyses will be completed in Review manager 5.3 (Cochrane, 2014). For categorical or dichotomous data, odds ratios with 95% confidence intervals will be calculated. For continuous data, standardised mean differences and 95% confidence intervals will be calculated. Appropriate data conversions will be made if necessary. Studies that reported range and appear skewed will be excluded in the meta-analysis.

**PROJECT TIMETABLE**

**REFERENCES**

Burazeri, G., Roshi, E., & Tavanxhi, N. (2004). Does knowledge about sexually transmitted infections increase the likelihood of consistent condom use? *Preventive Medicine*, *39*(6), 1077–9. doi:10.1016/j.ypmed.2004.04.016

Center For Disease Control and Prevention (CDC) (2008). *Sexually Transmitted Infections in Developing Countries; currrent concepts and strategies on improving STI treatment, prevention and control.*

CDC and The World Bank (2008). Sexually Transmitted Infections In Developing Countries: Current Concepts and Strategies in Improving STI Prevention, Treatment and Control. *A paper prepared by Team from both CDC and The World Bank.*

Centre for Reviews and Dissemination-University of York. *Data Extraction Form 2009*. Available at [www.crd.york.ac.uk/nihr_crweb/printpdf.php?ac](http://www.crd.york.ac.uk/nihr_crweb/printpdf.php?ac) (accessed on 12 April 2015).

Fonner, V. a, Armstrong, K. S., Kennedy, C. E., O’Reilly, K. R., & Sweat, M. D. (2014). School based sex education and HIV prevention in low- and middle-income countries: a systematic review and meta-analysis. *PloS One*, *9*(3), e89692. doi:10.1371/journal.pone.0089692

Gallant, M., & Maticka-Tyndale, E. (2004). School-based HIV prevention programmes for African youth. *Social Science & Medicine (1982)*, *58*(7), 1337–51. doi:10.1016/S0277-9536(03)00331-9

Gwet, K. (2002). Inter-Rater Reliability: Dependency on Trait Prevelance and Marginal Homgeneity. Statistical Methods for Inter-Rater Reliability Assessment Series, 2, 1-9.

Higgins JPT, Green S., ( editors). *Cochrane Handbook for Systematic Review of Interventions . 5.1.0 (update march 2011. The Cochrane Collabration, 2011.*  Available from : www.cochrane-handbook.org.

Higgins JPT, Green S., ( editors). *Cochrane Handbook for Systematic Review of Interventions . 5.1.0. The Cochrane Collabration, 2003.*  Available from : www.cochrane-handbook.org. Hipponi, J. A. C. (2003). METHODOLOGICAL INDEX FOR NON-RANDOMIZED STUDIES ( MINORS ): DEVELOPMENT AND VALIDATION OF A NEW INSTRUMENT, (May), 712–716.

Lindberg, L. D., & Maddow-Zimet, I. (2012). Consequences of sex education on teen and young adult sexual behaviors and outcomes. *The Journal of Adolescent Health : Official Publication of the Society for Adolescent Medicine*, *51*(4), 332–8. doi:10.1016/j.jadohealth.2011.12.028

Medley A., Kennedy C., O'Reilly K., & Sweat M. (2009). Effectiveness of Peer Education Interventions for HIV Prevention in Developing Countries. A systematic review and Meta-analysis. *AIDS Educ* Prev: *21*(3); 181-206. doi:10.1521/aeap.2009.21.3.181.Effectiveness

Michielsen, K., Chersich, M. F., Luchters, S., De Koker, P., Van Rossem, R., & Temmerman, M. (2010). Effectiveness of HIV prevention for youth in sub-Saharan Africa: systematic review and meta-analysis of randomized and nonrandomized trials. *AIDS (London, England)*, *24*(8), 1193–202. doi:10.1097/QAD.0b013e3283384791

Michielsen, K., Chersich, M., Temmerman, M., Dooms, T., & Van Rossem, R. (2012). Nothing as Practical as a Good Theory? The Theoretical Basis of HIV Prevention Interventions for Young People in Sub-Saharan Africa: A Systematic Review. *AIDS Research and Treatment*, *2012*, 345327. doi:10.1155/2012/345327

Napierala Mavedzenge, S. M., Doyle, A. M., & Ross, D. a. (2011). HIV prevention in young people in sub-Saharan Africa: a systematic review. *The Journal of Adolescent Health : Official Publication of the Society for Adolescent Medicine*, *49*(6), 568–86. doi:10.1016/j.jadohealth.2011.02.007

Paul-Ebhohimhen, V. a, Poobalan, A., & van Teijlingen, E. R. (2008). A systematic review of school-based sexual health interventions to prevent STI/HIV in sub-Saharan Africa. *BMC Public Health*, *8*, 4. doi:10.1186/1471-2458-8-4

Pienaar E., Gobler L., Busgeeth K., Eisinga A., Siegfried N. (2011). Developing a Geaographic Search Filter to Identify Randomised Controlled Trials in Africa. Finding the optional balance between sensitivity and precision. *Health Information and libraries Journal;* 28: 210-215. doi:10.1111/j1471-1842.2011.00936.x.

Ross D., Dick B., Ferguson J. (2006). Preventing HIV/AIDs in Young People. A Systematic Review of the Evidence from Developing Countries. *WHO Technical Report Series; no 938.*

Schaalma, H. P., Ph, D., Abraham, C., Gillmore, M. R., & Kok, G. (2004). Sex Education as Health Promotion : What Does It Take ?, *33*(3), 259–269.

The Cochrane Collaboration (2014).R eview Manager (RevMan) [Computer program]. Version 5.3. Copenhagen: The Nordic Cochrane Centre, The Cochrane Collaboration.

Wamoyi, J., Mshana, G., Mongi, A., Neke, N., Kapiga, S., & Changalucha, J. (2014). A review of interventions addressing structural drivers of adolescents’ sexual and reproductive health vulnerability in sub-Saharan Africa: implications for sexual health programming. *Reproductive Health*, *11*(1), 88. doi:10.1186/1742-4755-11-88

Wellings, K., Collumbien, M., Slaymaker, E., Singh, S., Hodges, Z., Patel, D., & Bajos, N. (2006). Sexual behaviour in context: a global perspective. *Lancet*, *368*(9548), 1706–28. doi:10.1016/S0140-6736(06)69479-8

WHO (2013). *Sexually Transmitted Infections (STIs)*. Available at: http://apps.who.int/iris/bitstream/10665/82207/1/WHO_RHR_13.02_eng.pdf (accessed 1 Decamber 2014).

WHO (2014). *Adolescent.* Available at: http://www.britannica.com/EBchecked/topic/6216/adolescence (accessed 19 February 2015)

Zhang, L., Hz, Q., Ml, B., Yin, L., Ruan, Y., & Sh, V. (2012). Internet-based behavioral interventions for preventing HIV infection in men who have sex with men ( MSM ) ( Protocol ), (12).

**APPENDIX**

Search Strategy for Medline

# Africa

1. ‘‘Africa’’[MeSH]
2. Africa*[tw]
3. Algeria[tw]
4. Angola[tw]
5. Benin[tw]
6. Botswana[tw]
7. ‘‘Burkina Faso’’[tw]
8. Burundi[tw]
9. Cameroon[tw]
10. ‘‘Canary Islands’’[tw]
11. ‘‘Cape Verde’’[tw]
12. ‘‘Central African Republic’’[tw]
13. Chad[tw]
14. Comoros[tw]
15. Congo[tw]
16. ‘‘Democratic Republic of Congo’’[tw]
17. Djibouti[tw]
18. Egypt[tw]
19. ‘‘Equatorial Guinea’’[tw]
20. Eritrea[tw]
21. Ethiopia[tw]
22. Gabon[tw]
23. Gambia[tw]
24. Ghana[tw]
25. Guinea[tw]
26. ‘‘Guinea Bissau’’[tw]
27. ‘‘Ivory Coast’’[tw]
28. ‘‘Cote d’Ivoire’’[tw]
29. Jamahiriya[tw]
30. Jamahiryia[tw]
31. Kenya[tw]
32. Lesotho[tw]
33. Liberia[tw]
34. Libya[tw]
35. Libia[tw]
36. Madagascar[tw]
37. Malawi[tw]
38. Mali[tw]
39. Mauritania[tw]
40. Mauritius[tw]
41. Mayote[tw]
42. Morocco[tw]
43. Mozambique[tw]
44. Mocambique[tw]
45. Namibia[tw]
46. Niger[tw]
47. Nigeria[tw]
48. Principe[tw]
49. Reunion[tw]
50. Rwanda[tw]
51. ‘‘Sao Tome’’[tw]
52. Senegal[tw]
53. Seychelles[tw]
54. ‘‘Sierra Leone’’[tw]
55. Somalia[tw]
56. ‘‘South Africa’’[tw]
57. ‘‘St Helena’’[tw]
58. Sudan[tw]
59. Swaziland[tw]
60. Tanzania[tw]
61. Togo[tw]
62. Tunisia[tw]
63. Uganda[tw]
64. ‘‘Western Sahara’’[tw]
65. Zaire[tw]
66. Zambia[tw]
67. Zimbabwe[tw]
68. ‘‘Central Africa*’’[tw]
69. ‘‘West* Africa*’’[tw]
70. ‘‘East* Africa*’’[tw]
71. ‘‘North* Africa*’’[tw]
72. ‘‘South* Africa*’’[tw]
73. ‘‘sub Saharan Africa*’’[tw]
74. ‘‘subSaharan Africa*’’[tw] or/ 1-74

# Adolescent

1. Adolescent/
2. Adolescen$.tw
3. Children/
4. Child*.tw
5. Teenager/
6. Teenager*.tw
7. Young Adults/
8. Young adult$.tw
9. Young people.tw
10. Youth$.tw
11. Student$.tw
12. Pupil$.tw
13. Schoolchildren.tw or /75-87

# School, sex and education

1. Schools/
2. Education/
3. Education.ti.
4. Teaching.ti.
5. Teach$.tw
6. School$.ti
7. School$.ab.
8. Curricul$.tw
9. (school$ adji5 sex$ adj5 (eduate$ or promot$ or intervene$ or teach$)).tw or/103-110
10. Sexually transmitted diseases/
11. HIV/
12. Acquired immunodeficiency Syndrome/
13. Sexual behave*r/
14. Sex/
15. Chlamydia infections/
16. Gonorrhoea/
17. Chlamydia trachomatis/
18. Syphilis/
19. Trichomoniasis/
20. Health education/
21. Health risk behaviour.tw
22. life style/
23. Social education.tw
24. Intervention strategy$.tw
25. Counsel$.tw
26. Health intervention$.tw
27. Social$develop$.tw
28. Education programme$.tw
29. Lifeskill$.tw
30. Resistance education.tw
31. Health education.tw
32. multiple partner$.ti,ab.
33. condom$.ti,ab. or condoms$.ti,ab.
34. (sex$ adj2 education$).tw
35. (sex$ adj3 transmit$ adj (disease$ or infection$)).tw or/88-122

#study design

1. Randomi?ed controlled trials/
2. Controlled clinical trials/
3. Double-blind studies/
4. Single-blind studies/
5. Follow-up studies/
6. Comparative studies/
7. Evaluation studies/
8. Intervention studies/
9. Multicentre studies/
10. Program evaluation/
11. Case control studies/
12. Pilot studies/
13. Validation studies/
14. Random$.tw Or/123-136.
