# Supplementary material for: School-based sexual health education interventions to prevent STI/HIV in sub-Saharan Africa: a systematic review and meta-analysis
Source: BMC Public Health. 2016 Oct 10;16:1069. doi: 10.1186/s12889-016-3715-4 (PMC5057258; doi:10.1186/s12889-016-3715-4)
Supplement: Additional file 2: — Search Strategy for Medline Which Was Modified and Used In Other Databases. (DOCX 21 kb) [file 12889_2016_3715_MOESM2_ESM.docx]

**SUPPLEMENTARY FILE 2: Search Strategy for Medline Which Was Modified and Used In Other Databases**.

| # Africa  1. ‘‘Africa’’ [MeSH]  2. Africa*[tw]  3. Algeria [tw]  4. Angola [tw]  5. Benin [tw]  6. Botswana [tw]  7. ‘‘Burkina Faso’’ [tw]  8. Burundi [tw]  9. Cameroon [tw]  10. ‘‘Canary Islands’’ [tw]  11. ‘‘Cape Verde’’ [tw]  12. ‘‘Central African Republic’’ [tw]  13. Chad [tw]  14. Comoros [tw]  15. Congo [tw]  16. ‘‘Democratic Republic of Congo’’ [tw]  17. Djibouti [tw]  18. Egypt [tw]  19. ‘‘Equatorial Guinea’’ [tw]  20. Eritrea [tw]  21. Ethiopia [tw]  22. Gabon [tw]  23. Gambia [tw]  24. Ghana [tw]  25. Guinea [tw]  26. ‘‘Guinea Bissau’’ [tw]  27. ‘‘Ivory Coast’’ [tw]  28. ‘‘Cote d’Ivoire’’ [tw]  29. Jamahiriya [tw]  30. Jamahiriya [tw]  31. Kenya [tw]  32. Lesotho [tw]  33. Liberia [tw]  34. Libya [tw]  35. Libya [tw]  36. Madagascar [tw]  37. Malawi [tw]  38. Mali [tw]  39. Mauritania [tw]  40. Mauritius [tw]  41. Mayotte [tw]  42. Morocco [tw]  43. Mozambique [tw]  44. Mozambique [tw]  45. Namibia [tw]  46. Niger [tw]  47. Nigeria [tw]  48. Principe [tw]  49. Reunion [tw]  50. Rwanda [tw]  51. ‘‘Sao Tome’’ [tw]  52. Senegal [tw]  53. Seychelles [tw]  54. ‘‘Sierra Leone’’ [tw]  55. Somalia [tw]  56. ‘‘South Africa’’ [tw]  57. ‘‘St Helena’’ [tw]  58. Sudan [tw]  59. Swaziland [tw]  60. Tanzania [tw]  61. Togo [tw]  62. Tunisia [tw]  63. Uganda [tw]  64. ‘‘Western Sahara’’[tw]  65. Zaire[tw]  66. Zambia[tw]  67. Zimbabwe[tw]  68. ‘‘Central Africa*’’[tw]  69. ‘‘West* Africa*’’[tw]  70. ‘‘East* Africa*’’[tw]  71. ‘‘North* Africa*’’[tw]  72. ‘‘South* Africa*’’[tw]  73. ‘‘sub Saharan Africa*’’[tw]  74. ‘‘subSaharan Africa*’’ [tw] or/ 1-74  # Adolescent  75. Adolescent/  76. Adolescen$.tw  77. Children/  78. Child*.tw  79. Teenager/  80. Teenager*.tw  81. Young Adults/  82. Young adult$.tw  83. Young people.tw  84. Youth$.tw  85. Student$.tw  86. Pupil$.tw  87. Schoolchildren.tw or /75-87  # School, sex and education, sexually transmitted infection.  88. Schools/  89. Education/  90. Education.ti.  91. Teaching.ti.  92. Teach$.tw  93. School$.ti  94. School$.ab.  95. Curricul$.tw  96. (school$ adji5 sex$ adj5 (eduate$ or promot$ or intervene$ or teach$)).tw  97. Sexually transmitted diseases/  98. HIV/  99. Acquired immunodeficiency Syndrome/  100. Sexual behave*r/  101. Sex/  102. Chlamydia infections/  103. Gonorrhoea/  104. Chlamydia trachomatis/  105. Syphilis/  106. Trichomoniasis/  107. Health education/  108. Health risk behaviour.tw  109. life style/  110. Social education.tw  111. Intervention strategy$.tw  112. Counsel$.tw  113. Health intervention$.tw  114. Social$develop$.tw  115. Education programme$.tw  116. Lifeskill$.tw  117. Resistance education.tw  118. Health education.tw  119. multiple partner$.ti,ab.  120. condom$.ti,ab. or condoms$.ti,ab.  121. (sex$ adj2 education$).tw  122. (sex$ adj3 transmit$ adj (disease$ or infection$)).tw or/88-122  #study design  123. Randomi?ed controlled trials/  124. Controlled clinical trials/  125. Double-blind studies/  126. Single-blind studies/  127. Follow-up studies/  128. Comparative studies/  129. Evaluation studies/  130. Intervention studies/  131. Multicentre studies/  132. Program evaluation/  133. Case control studies/  134. Pilot studies/  135. Validation studies/  136. Random$.tw Or/123-136. |
| --- |
