# Supplementary material for: School-based sexual health education interventions to prevent STI/HIV in sub-Saharan Africa: a systematic review and meta-analysis
Source: BMC Public Health. 2016 Oct 10;16:1069. doi: 10.1186/s12889-016-3715-4 (PMC5057258; doi:10.1186/s12889-016-3715-4)
Supplement: Additional file 3: — Data Extraction Form. (DOCX 26 kb) [file 12889_2016_3715_MOESM3_ESM.docx]

# SUPPLEMENTARY FILE 3: Data Extraction Form

| **LEAD AUTHOR’S NAME** |  |
| --- | --- |
| **DATE OF PUBLICATION** |  |
| **TITLE OF ARTICLE** |  |
| **TYPE OF PUBLICATION** (Journal, conference abstracts etc.) |  |
| **JOURNAL TITLE** |  |
| **COUNTRY OF INTERVENTION** |  |
| **SOURCE OF FUNDING** |  |
| **STUDY AIMS AND OBJECTIVES** |  |
| **SCHOOL TYPE** |  |
| **DEMORGRAHIC OF PARTICIPANTS**  **(AGE, SEX , CLASS/GRADE)** |  |
| **STUDY DESIGN** |  |
| **DESCRIPTION OF INTERVENTION** |  |
| **DETAILS OF INTERVENTION**   1. Frequency/dose. 2. Duration. 3. Year(s) of intervention. 4. Theory or theories used. |  |
| **DETAILS OF CONTROL** |  |
| **DETAILS OF IMPLEMENTATION.**   1. Who delivered the intervention? 2. Was the instructor(s) trained? 3. Was the implementation monitored? 4. Was the intervention delivered as designed? 5. Challenges encountered during the implementation. |  |
| **OUTCOMES**   1. Primary outcomes  - Condom use - Prevalence of STI/HIV/unwanted pregnancy.  1. Was the measurement objective or subjective (self-reported or investigator reported)? 2. Length of follow-up. |  |
| **RESULTS**   1. Statistical technique(s) used 2. Number of participants  - Baseline/pre-intervention - Follow-up - Number of loss to follow-up/withdrawals  1. Summary of results.   For each outcome reported,   - Baseline - Follow-up |  |
| **AUTHOR’S KEY CONCLUSION** |  |

THE COCHRANE COLLABRATION’S TOOL FOR ASSESSING RISK OF BIAS

| Domain | Evidence | Risk and score |
| --- | --- | --- |
| SELECTION BIAS   - Random sequence generation. - Allocation concealment. - Bias due to confounding |  |  |
| PERFORMANCE BIAS   - Blinding of participants and personnel. |  |  |
| DETECTION BIAS   - Blinding of outcome assessment |  |  |
| ATTRITION BIAS   - Incomplete outcome data. |  |  |
| REPORTING BIAS   - Selective reporting |  |  |
| OTHER SOURCES OF BIAS |  |  |

Overall score___________

| Characteristics of effective interventions | Evidence |
| --- | --- |
| DESIGN RELATED   - Need assessment and involvement of key stakeholders. - Adapting from other programs or curriculum that are found to be efficacious. - Theory-based. - Skilled-based. |  |
| IMPLEMENATION RELATED   - Provision of adolescents health services. - Distribution of condoms. - Activities outside school environment. - Training of facilitators. - Implementation of intervention with fidelity. |  |
