# Supplementary material for: School-based sexual health education interventions to prevent STI/HIV in sub-Saharan Africa: a systematic review and meta-analysis
Source: BMC Public Health. 2016 Oct 10;16:1069. doi: 10.1186/s12889-016-3715-4 (PMC5057258; doi:10.1186/s12889-016-3715-4)
Supplement: Additional file 4: — Lists of Excluded Studies with Reasons for the Exclusion. (DOCX 28 kb) [file 12889_2016_3715_MOESM4_ESM.docx]

**SUPPLEMENTARY FILE 4: Lists of Excluded Studies with Reasons for the Exclusion.**

| 1. Aaro et al 2014: a study protocol. 2. Adeoam 2012: delivered in university. 3. Adam et al 2014: intervention delivered in university. 4. Adegbenro 2014: the participants are teachers (Conference abstract). 5. Adegbenro et al 2013: conference abstract and full article not obtained. 6. Adeomi et al 2014: outcome not of interest. 7. Adibe 2013: PhD thesis full text not available. 8. Agha 2001: setting not school. 9. Agha 2002: intervention reported in already included paper. 10. Agha 2002: the setting is not school. 11. Ahmed 2006: teachers training are evaluated. 12. Ahmed et al 2007: educators’ belief is assessed. 13. Ahmed et al 2009: teachers’ survey. 14. Ajewole and Osagbane 2007: intervention in rural community not school. 15. Ajewole and Osagbemi 2007: The setting is youth centre (not school). 16. Akpabio et al 2009: outcome not of interest. 17. Amuago et al 2014: a systematic review. 18. Andrew 2001: intervention not delivered in school. 19. Arinze-onyia 2013: outcome measure is Knowledge and use of Emergency contraception. 20. Armistead et al 2014: intervention delivered at homes. 21. Baldo et al 2000: indicators developments only discussed in the paper. 22. Baptiste et al 2006: setting is not school. 23. Bastien et al 2008. the design is cross sectional. 24. Batist et al 2013: the setting is not school and participants are men who have sex with men (MSM). 25. Bekele and Ali 2008: pre and post-test study design. 26. Belle et al 2010: theory driven evaluation design article. 27. Bhana 2005: a power point presentation for conference. 28. Bing 2008. the participants are soldiers and setting is military base. 29. Bjorkman-Nyquist 2013: the setting is not school (conference poster). 30. Bogale et al, 2011: not in school setting. 31. Borgat et al 2011: setting is workplace and participants are parents of adolescents. 32. Brown et al 2008: setting is university. 33. Buhari et al 2012: outcome not of interest. 34. Caldwell et al 2004: curriculum development no outcome measured. 35. Cameron et al 2014: setting not schools. 36. Cameron Wolf et al 2000: not an intervention. 37. Cameronwolf et al 2002: measured number and characteristics of peer educators and their contacts. 38. Campbell and MacPhail, 2002: a longitudinal case study. 39. Card et al 2011: a description (not an intervention). 40. Chifunyise et al 2002: the intervention target school teachers. 41. Clark et al 2006: outcome not of interest. 42. Cluver et al 2013: a case-control study. 43. Cluver et al 2014: a prospective observational study. 44. Coffman et al: no control group (before and after intervention design). 45. Cowan et al 2002: evaluation studies. 46. Coyle et al 2012: setting not Africa. 47. Creese et al 2002: a cost effectiveness analysis. 48. Diane et al 2009: conference PowerPoint slides and full text article not obtained. 49. DiClemente and Crosby 2009: a report (Not evaluated intervention). 50. DiClemente et al 2014: setting not Africa. 51. DiIorio et al 2007: setting not Africa. 52. Doyles et al 2010: intervention reported in already included paper. 53. Duflo et al 2006: before and after intervention. 54. Dunbar et al 2010: setting not school. 55. Dunbar et al 2014: not in school setting. 56. Duong 2007: a cross sectional study. 57. Dupas et al 2012: conference abstract full text not accessible. 58. Eberso¨hn et al 2011: a longitudinal study. 59. Enah et al 2010: before and after study. 60. Epstein, et al 2010: a correspondence. 61. Erulka et al 2004: not in school setting. 62. Exner et al 2006: conference abstract and full text not obtained. 63. Fagen et al 2006: setting not Africa. 64. Feldman et al 1997: a cross sectional survey. 65. Fitzgerald et al 1999: outcome not of interest. 66. Foluso and odu 2010: intervention delivered in university. 67. Foss et al 2007: systematic review. 68. Fuller et al 2007: outcome measured not of interest. 69. Gallant and Maticka-Tyndale, 2004: systematic review. 70. Goesling et al 2014: systematic review. 71. Goeslins et al 2013: a systematic review. 72. Goldstein et al 2005: the setting not school. 73. Gudyanga et al 2013: a cross sectional survey. 74. Haglund 2008: setting is not Africa. 75. Hallman et al 2007: outcome not of interest. 76. Halfors et al 2015: Intervention not sex education. 77. Harrison 2004: intervention delivered during rural outreach. 78. Harrison et al 2010: a systematic review. 79. Hayes et al 2005: intervention reported in already included paper. 80. Hayes et al 2005: a description of intervention no outcome measured. 81. Heeren et al 2013: delivered in universities. 82. Helle et al 2013: intervention delivered in university. 83. Helleve et al 2011: a secondary data analysis. 84. Helpern et al 2008: outcome not of interest. 85. Hennin 2010: a cross sectional study. 86. Hermanns et al 2009: no control arm i.e. pre and post-test design. 87. Hervey et al 2000: the control group received a form of sex education. 88. Hogan et al 2005: a cost effectiveness analysis. 89. Hope 2010: the setting is workplace. 90. Horizon et al 2008: before and after design no comparison group. 91. Hosek, et al 2011: setting not Africa. 92. Irvin 2000: a discussion paper. 93. James Jacob et al 2007: a cross sectional secondary data analysis. 94. Jansen Van Rensburg 2007: the setting is not school. 95. Jemmott III et al 2010: setting not Africa. 96. Jemmott III et al, 2014: setting not school. 97. Jensen Van Rendurg 2007: outcome measured is gender based violence. 98. Jewkes et al 2006: rural communities (not school setting). 99. Jewkes et al 2008: intervention delivered in villages (not school setting). 100. Jewkes et al 2010: an opinion paper. 101. Jukes et al 2008: not an intervention. 102. Kaaya et al 2002: a systematic review. 103. Kafewo 2008: a description of an intervention. 104. Kajubi, et al 2005: setting not school. 105. Kalichman et al 2009: setting is not school. 106. Kamali et al 2002: not in school setting. 107. Katsinde et al 2011: a descriptive cross sectional survey. 108. Kaufman et al 2012: not in Africa. 109. Kaufman et al 2013: a conference poster and full article not obtained. 110. Kaufman et al 2013: a systematic review. 111. Keating et al 2006: intervention not in school. 112. Kellam et al 2008: the setting is Baltimore, USA. 113. Kennedy 2012: conferences abstract and the full article not obtained. 114. Kennedy et al 2012: reported only baseline data. 115. Kennedy et al 2014: a systematic review. 116. Kent et al 2005: intervention target health science students and measure skills towards care for HIV patients. 117. Key et al 2008: setting not Africa. 118. Kim et al 2001: not in school setting. 119. Kimani et al 2012: before and after interventions. 120. Kinsman et al 2001: outcome not of interest. 121. Kirby et al 2008: a systematic review. 122. Klepp et al 1997: outcome not of interest. 123. Kuhn et al 1994: outcome not of interest. 124. Kumakech et al 2009: the outcome is psychological well-being. 125. Laneri 1996: intervention delivered in America. 126. Larke et al 2010: outcome measured is utilization of health services. 127. Lemma et al 2008: a cross sectional study. 128. Lightfoot et al 2007: not school setting. 129. Lupiwa et al 1996: a cross sectional survey. 130. Maclachlan et al 1997: outcome not of interest. 131. MacPhail et al 2013: outcome not of interest. 132. Madeni et al 2011: no comparison group in the study. 133. Magid et al 1998: delivered at homes or religious gatherings. 134. Magnani et al 20005: before and after intervention. 135. Mantell et al 2014: the control arm also receives a form of sex education 136. Maro et al 2009: outcome not of interest. 137. Maro et al 2009: setting is a youth centre though comparison group are in-school youths. 138. Mash et al 2012: setting is a church congregation. 139. Mason-Jones et al 2011: not an intervention. 140. Massey et al : outcome not of interest. 141. Mathews et al 1996: before and after intervention. 142. Maticka-Tyndale 2004: outcome not of interest. 143. Mbizo et al 1997: outcome not of interest. 144. Mbonye 2003: a cross sectional survey. 145. McCreary et al 2010: setting not school. 146. McCree-Hale et al 2012: a formative research and is a poster. 147. Meekers et al 2005: before and after intervention. 148. Michielsen et al 2012: a systematic review. 149. Michielsena et al 2010: a systematic review. 150. Miller et al 2008: pre and post-test, no control group. 151. Mitchell et al 2007: participants are out of school youths. 152. Mkumbo et al 2009: a description of intervention development. 153. Munodawafa et al 1995: outcome not of interest. 154. Muyinda et al 2004: setting not school. 155. Mwale 2008: a cross sectional survey. 156. Ndeki et al 1994: a cross sectional survey. 157. NIMS trial group 2010: the setting is not school. 158. Njau et al 2006: a descriptive cross sectional. 159. Njue et al 2009: pre and post, no control group. 160. Nyawasha et al 2013: setting not school. 161. O’donnell et al 2002: setting not Africa. 162. O’leary et al 2012: intervention already reported in another included study. 163. O'Donnell et al 1999: setting not Africa. 164. Odundo et al 2013: meeting abstract and full text not obtained. 165. Okonkwo et al 2009: full article not obtained although request send to the author. 166. Ower et al 2004: an intervention description. 167. Paul-Ebhohimhen et al 2008: a systematic review. 168. Peltzer et al 2005: teachers’ perspective studied. 169. Picot et al 2012: a systematic review. 170. Power et al 2004: no outcome measured (a description of intervention). 171. Quigley et al 2004: not in school setting. 172. Quirk et al 1993: intervention delivered in a health centre. 173. Reddy 2005: outcome not of interest. 174. Rotheram-Borus et al 2012: setting is a youth centre. 175. Saad et al 2012: delivered in universities. 176. Sabage et al 2014: a cross sectional survey. 177. Saca et al 2005: a cross sectional Survey. 178. Sacadura et al 2005: the article evaluates teachers’ perception of the intervention. 179. sandøy et al 2012: setting not schools. 180. Scott-Sheldon et al 2013: a systematic review. 181. Sekoni et al: setting not school. 182. Sherman et al 1999: a cross sectional survey. 183. Shuey et al 1999: outcome not of interest. 184. Siegel et al 1995: intervention delivered in California, USA. 185. Siegel et al 1998: setting is not Africa. 186. Simonelli et al 2002: conference abstract and full text not obtained. 187. Small et al 2013: a systematic review. 188. Smit et al 2012: a conference poster and full article not obtained. 189. Speizer et al 2001: intervention delivered in communities not in school setting. 190. Ssewamala et al 2010: outcome not of interest. 191. Stadler and Hlongwa 2002: setting not school. 192. Stigler et al 2006: outcome not of interest. 193. Stroeken et al 2012: a systematic review. 194. Sukati et al 2010: a descriptive study design. 195. Sweat et al 2011: setting not school. 196. Tindale et al 1997: health education not sex education delivered. 197. Todd et al 2013: a comparison of efficiency of different trial design. 198. Tortolero et al 2008: setting not Africa. 199. Townsend et al 2013: a systematic review. 200. Underwood etr al 2005: setting not school. 201. Unicef Ghana 2002: setting not in school and no matched control. 202. Van der Straten et al 2010: setting not schools. 203. Van Reeuwijk 2009: not an intervention. 204. Van Rossem and Meekers 1999: setting not school. 205. Van-Velthoven et al 2013: a systematic review. 206. Visser 2005: before and after study. 207. Visser 2007: before and after study. 208. Visser et al 2004: before and after study. 209. Wanyama et al 2012: a clinic setting. 210. Wawer et al 1998: community setting (not school). 211. Wawer et al 1999: intervention not in school. 212. Wight et al 2012: intervention already included in another included study. 213. Wingood et al 2006: setting not Africa. 214. Wingood et al 2013: setting not schools. 215. Witte et al 1998: setting not schools. 216. Yankah et al 2008: a systematic review. 217. Ybarra et al 2013: the control group also receive a form of sex education. 218. Yotebieng et al 2009: a cross-sectional secondary data analysis. 219. Zani et al 2011: a systematic review. 220. Zuch et al 2012: not an intervention. |
| --- |
