# Supplementary material for: School-based sexual health education interventions to prevent STI/HIV in sub-Saharan Africa: a systematic review and meta-analysis
Source: BMC Public Health. 2016 Oct 10;16:1069. doi: 10.1186/s12889-016-3715-4 (PMC5057258; doi:10.1186/s12889-016-3715-4)
Supplement: Additional file 5: — Modified Cochrane Collaboration Tool for Assessing Risk of Bias. (DOCX 45 kb) [file 12889_2016_3715_MOESM5_ESM.docx]

**SUPPLEMENTARY FILE 5: Modified Cochrane Collaboration Tool for Assessing Risk of Bias**

| Study |  | Selection Bias |  |  | Performance Bias | Detection Bias | Attrition  Bias | Overall Score (Risk) |
| --- | --- | --- | --- | --- | --- | --- | --- | --- |
|  |  | Random Sequence Generation | Allocation Concealment | Bias Due to Confounding | Blinding of Participants and Personnel | Blinding of Outcome Assessment | Incomplete Outcome Data |  |
| Aderibigbe and Araoye 2008 | | Not applicable | Not applicable | No statistically significant difference in the distribution between the study and the control group in age, sex, ethnic group or religion (Low) (0) | Not practical (Low) | Not practical (Low) | Number of loss to follow up was not reported (Unclear) (1) | 1 (Low) |
| Agha &Rossem 2004 | | "…using a random generation process…" (Low) (0) | Not reported (unclear) | Not applicable | Not practical (Low) | Not practical (Low) | The students missed at follow up do not resume in the term assessment was made and follow up rate is 86% (Low) (0) | 0 (Low) |
| Ajuwon and Brieger 2007 | | Not applicable | Not applicable | There was statistically significant difference between the two arms of the study in age, baseline knowledge score for reproductive health which were not accounted for in the analysis (High) (2) | Not practical (Low) | Not practical (Low) | The number rose at follow up and explanation not given (unclear) (1) | 3 (High) |

**SUPPLEMENTARY FILE 5: Modified Cochrane Collaboration Tool for Assessing Risk of Bias Cont.**

| Study |  | Selection Bias |  |  | Performance Bias | Detection Bias | Attrition  Bias | Overall Score (Risk) |
| --- | --- | --- | --- | --- | --- | --- | --- | --- |
|  |  | Random Sequence Generation | Allocation Concealment | Bias Due to Confounding | Blinding of Participants and Personnel | Blinding of Outcome Assessment | Incomplete Outcome Data |  |
| Arnold et al 2012 | | The schools were randomly selected details not given (Unclear) (1) | Not reported (Low) | Not applicable | Not practical (Low) | Not practical (Low) | Number increased due to education reform in the state that encourage school enrolment (Low) (0) | 1 (Low) |
| Atwood et al 2012 | | Randomisation mentioned but details not given (Unclear) (1) | Not practical (Low) | Not applicable | Not practical (Low) | Not practical (Low) | 88% of the participants at baseline were reached at follow up (Low) (0) | 1 (Low) |
| Brieger et al 2001 | | Not applicable | Not applicable | "…results showed gender differences in the total number of youth reached. Additional analysis was performed among the intervention youth at follow up to examine gender differences…" (Low) (0) | Not practical (Low) | Not practical (Low) | Loss to follow up not reported (Unclear) (1) | 1 (Low) |

**SUPPLEMENTARY FILE 5: Modified Cochrane Collaboration Tool for Assessing Risk of Bias Cont.**

| Study |  | Selection Bias |  |  | Performance Bias | Detection Bias | Attrition  Bias | Overall Score (Risk) |
| --- | --- | --- | --- | --- | --- | --- | --- | --- |
|  |  | Random Sequence Generation | Allocation Concealment | Bias Due to Confounding | Blinding of Participants and Personnel | Blinding of Outcome Assessment | Incomplete Outcome Data |  |
| Burnett et al 2011 | | Not reported (unclear) (1) | Not reported (unclear) | Not applicable | Not practical (Low) | Not practical (Low) | Loss to follow up is around 20% in each arm. Reason for attrition given which was statistically significantly different between the intervention and control group (Low) (0) | 1 (Low) |
| Cowan et al 2010 | | Not reported (unclear) (1) | Not reported(Low) | Not applicable | Not practical (Low) | Not practical (Low) | Not reported (Unclear) (1) | 2 (Moderate) |
| Cupp et al 2008 | | Not reported (unclear) (1) | Not reported (unclear) | Not applicable | Not practical (Low) | Not practical (Low) | Number lost to follow up not reported or accounted for in the analysis (Unclear) (1) | 2 (Moderate) |

**SUPPLEMENTARY FILE 5: Modified Cochrane Collaboration Tool for Assessing Risk of Bias Cont.**

| Study |  | Selection Bias |  |  | Performance Bias | Detection Bias | Attrition  Bias | Overall Score (Risk) |
| --- | --- | --- | --- | --- | --- | --- | --- | --- |
|  |  | Random Sequence Generation | Allocation Concealment | Bias Due to Confounding | Blinding of Participants and Personnel | Blinding of Outcome Assessment | Incomplete Outcome Data |  |
| Denison et al 2012 | | Not applicable | Not applicable | No statistically significant difference between the intervention and control group in age, gender, religion, current grade, orphan status and living situation (Low) (0) | Not practical (Low) | Not practical (Low) | Reason for attrition reported and loss to follow up is around 14% in both arm of the intervention (Low) (0) | 0 (Low) |
| Esere 2008 | | Not applicable | Not applicable | Age, gender, and pre-test scores are used as covariates (Low) (0) | Not practical (Low) | Not practical (Low) | All participants retained at follow up (low) (0) | 0 (Low) |
| Fawole et al 1999 | | Simple balloting used for randomisation (low) (0) | Not reported (unclear) | Not applicable | Not practical (Low) | Not practical (Low) | Reported but reason not given but loss to follow up is 3.8% in both arms (low) (0) | 0 (Low) |

**SUPPLEMENTARY FILE 5: Modified Cochrane Collaboration Tool for Assessing Risk of Bias Cont.**

| Study |  | Selection Bias |  |  | Performance Bias | Detection Bias | Attrition  Bias | Overall Score (Risk) |
| --- | --- | --- | --- | --- | --- | --- | --- | --- |
|  |  | Random Sequence Generation | Allocation Concealment | Bias Due to Confounding | Blinding of Participants and Personnel | Blinding of Outcome Assessment | Incomplete Outcome Data |  |
| James et al 2005 | | Not reported (unclear) (1) | Not reported (unclear) | Not applicable | Not practical (Low) | Not practical (Low) | The dropout rate was 38.2% which was due to impending school examinations in both arms of the study. (High) (2) | 3 (High) |
| James et al 2006 | | Not applicable | Not applicable | Age, gender, and language do not differ between the two arms but religion which was not included as an additional predictor in the design owing to the low count of non-Christians in certain schools. (Low) (0) | Not practical (Low) | Not practical (Low) | Dropout rate is 32.7%. (High) (2) | 2 (Moderate) |
| Jemmott et al 2015 | | Computer generated number sequence used (Low) (0) | “ …using concealment allocation techniques to minimizes bias …” (Low) | Not applicable | Not practical (Low) | Not practical (Low) | Intention to treat analysis performed and loss to follow up is less than 10% in each arm of the study (Low) (0) | 0 (Low) |

**SUPPLEMENTARY FILE 5: Modified Cochrane Collaboration Tool for Assessing Risk of Bias Cont.**

| Study |  | Selection Bias |  |  | Performance Bias | Detection Bias | Attrition  Bias | Overall Score (Risk) |
| --- | --- | --- | --- | --- | --- | --- | --- | --- |
|  |  | Random Sequence Generation | Allocation Concealment | Bias Due to Confounding | Blinding of Participants and Personnel | Blinding of Outcome Assessment | Incomplete Outcome Data |  |
| Karnell et al 2006 | | Not applicable | Not applicable | Age was the only socio demographic factor found to differ between the two arms of the study and so controlled for in the analysis (Low) (0) | Not practical (Low) | Not practical (Low) | Reported and accounted for in the analysis. Loss to follow up is less than 20% (Low) (0) | 0 (Low) |
| Mason-Jones et al 2011 | | Not applicable | Not applicable | There was imbalance between the two arms of the study in socio-economic characteristics. Age, racially social group, baseline proportion and cluster were adjusted for in the analysis. (Low) (0) | Not practical (Low) | Not practical (Low) | Reason for loss to follow up given. Loss to follow up is 41.9% in the intervention arm and 36.4% in the control arm (High) (2) | 2 (Moderate) |
| Mason-Jones et al 2013 | | Not applicable | Not applicable | Participants were fairly matched between the two arms on socio-economic characteristics. Baseline and clustering were adjusted in the analysis (Low) (0) | Not practical (Low) | Not practical (Low) | Reason for attrition given and is equal for both intervention and control group. Loss to follow up is 30.6% (High) (2) | 2 (Moderate) |

**SUPPLEMENTARY FILE 5: Modified Cochrane Collaboration Tool for Assessing Risk of Bias Cont.**

| Study |  | Selection Bias |  |  | Performance Bias | Detection Bias | Attrition  Bias | Overall Score (Risk) |
| --- | --- | --- | --- | --- | --- | --- | --- | --- |
|  |  | Random Sequence Generation | Allocation Concealment | Bias Due to Confounding | Blinding of Participants and Personnel | Blinding of Outcome Assessment | Incomplete Outcome Data |  |
| Mathews et al 2012 | | "In Cape Town and Merkweng, one school in each pair was randomly allocated to the intervention arm of the study by putting the school names in a container, one pair at a time and drawing the one to be the intervention school" and random number table was used in Dar es slam (Low) (0) | Not reported (unclear) | Not applicable | Not practical (Low) | Not practical (Low) | Reason reported and the loss to follow up is less than 30% in each arm in all the three sites. (Low) (0) | 0 (Low) |
| Maticka-Tyndale et al 2007 | | Not applicable | Not applicable | Some demographic variables were different between the two arms of the study. However, all demographic variables, pre-post and control-intervention were controlled for in the analysis (Low) (0) | Not practical (Low) | Not practical (Low) | Number seems to increase at follow up and reason not given (unclear) (1) | 1 (Low) |

**SUPPLEMENTARY FILE 5: Modified Cochrane Collaboration Tool for Assessing Risk of Bias Cont.**

| Study |  | Selection Bias |  |  | Performance Bias | Detection Bias | Attrition  Bias | Overall Score (Risk) |
| --- | --- | --- | --- | --- | --- | --- | --- | --- |
|  |  | Random Sequence Generation | Allocation Concealment | Bias Due to Confounding | Blinding of Participants and Personnel | Blinding of Outcome Assessment | Incomplete Outcome Data |  |
| Mba et al 2007 | | Schools were selected by basket methods of random sampling (Low) (0) | Not reported (unclear) | Not reported (Unclear) | Not practical (Low) | Not practical (Low) | Not reported (Unclear) (1) | 1 (Low) |
| Menna et al 2015 | | Not applicable | Not applicable | Socio demographic characteristics like sex, age, religion and ethnicity were controlled for in the analysis (Low) (0) | Not practical (Low) | Not practical (Low) | Loss to follow ups were 7.9% in the control group and zero percent in the intervention group (Low) (0) | 0 (Low) |
| Michielsen et al 2012 | | Not applicable | Not applicable | Adjustment for propensity score was made and included in the analysis (Low) (0) | Not practical (Low) | Not practical (Low) | Reason given and accounted for in the analysis. Retention rate is 71.8% at follow up (Low) (0) | 0 (Low) |

**SUPPLEMENTARY FILE 5: Modified Cochrane Collaboration Tool for Assessing Risk of Bias Cont.**

| Study |  | Selection Bias |  |  | Performance Bias | Detection Bias | Attrition  Bias | Overall Score (Risk) |
| --- | --- | --- | --- | --- | --- | --- | --- | --- |
|  |  | Random Sequence Generation | Allocation Concealment | Bias Due to Confounding | Blinding of Participants and Personnel | Blinding of Outcome Assessment | Incomplete Outcome Data |  |
| Okonofua et al 2003 | | Not reported (unclear) (1) | Not reported (unclear) | Not applicable | Not practical (Low) | Not practical (Low) | Not reported (Unclear) (1) | 2 (Moderate) |
| Rijsdijk et al 2011 | | Not applicable | Not applicable | Age, gender, and control/intervention variables were used as covariates (low) (0) | Not practical (Low) | Not practical (Low) | Reason given and removed from the analysis. Loss to follow up is less than 30% in each arm of the study (Low) (0) | 0 (Low) |
| Ross et al 2007 | | A computer programme was used for randomisation (Low) (0) | Not reported (unclear) | Not applicable | Not practical (Low) | Not practical (Low) | Reason for attrition given and appeared to be the same for both groups and more than 70% of participants were retained at follow up (Low) (0) | 0 (Low) |

**SUPPLEMENTARY FILE 5: Modified Cochrane Collaboration Tool for Assessing Risk of Bias Cont.**

| Study |  | Selection Bias |  |  | Performance Bias | Detection Bias | Attrition  Bias | Overall Score (Risk) |
| --- | --- | --- | --- | --- | --- | --- | --- | --- |
|  |  | Random Sequence Generation | Allocation Concealment | Bias Due to Confounding | Blinding of Participants and Personnel | Blinding of Outcome Assessment | Incomplete Outcome Data |  |
| Stanton et al 1998 | | "…randomly assigned using study identification number and a random numbers table" (low) (0) | Not reported (unclear) | Not applicable | Not practical (Low) | Not practical (Low) | Reason given and similar in each groups and 70% of the participants were retained at follow up (Low) (0) | 0 (Low) |
| Taylor et al 2014 | | Not reported (unclear) (1) | Not reported (unclear) | Not applicable | Not practical (Low) | Not practical (Low) | Dropout rate is 16.6% and is more in the control group, details not given (Unclear) (1) | 2 (Moderate) |

**SUPPLEMENTARY FILE 5: Modified Cochrane Collaboration Tool for Assessing Risk of Bias Cont.**

| Study |  | Selection Bias |  |  | Performance Bias | Detection Bias | Attrition  Bias | Overall Score (Risk) |
| --- | --- | --- | --- | --- | --- | --- | --- | --- |
|  |  | Random Sequence Generation | Allocation Concealment | Bias Due to Confounding | Blinding of Participants and Personnel | Blinding of Outcome Assessment | Incomplete Outcome Data |  |
| Tibbits et al 2011 | | Randomly selected but no details (Unclear) (1) | Not reported (unclear) | Not applicable | Not practical (Low) | Not practical (Low) | Attrition was accounted for in the calculation. Attrition rate is about 38% at the final follow up (High) (2) | 3 (High) |
| Van der Maas and Otte 2009 | | Not applicable | Not applicable | Age, sex, and residence are the confounders accounted for in the analysis (Low) (0) | Not practical (Low) | Not practical (Low) | Not reported (Unclear) (1) | 1 (Low) |
| Ybarra et al 2013 | | "youth were then randomly selected by the research team using randomizer.org" (low) (0) | “Randomization to the intervention or control arm was executed using code embedded in the software program…” (low) | Not applicable | Not practical (Low) | Not practical (Low) | Retention rate is 93% at final follow up (Low) (0) | 0 (Low) |

Note:

1. Selection bias due to confounding: A study was classified as ‘low risk’ of bias if it assessed the balance of confounders between the two arms of the study and controlled for them in the analysis if not balanced.
2. Incomplete outcome data: We use an attrition rate of not more than 30% in each arm of the study at follow up to classify as ‘low risk’ of bias for the dimension of incomplete outcome data. Attrition rates of 30% and 40% are the cut off thresholds recommended for qualifying scientific rigour of effective and promising evidenced-based behavioural interventions respectively *.
3. All the remaining dimensions were assessed as described in The Cochrane Collaboration Tool for Assessing Risk of Bias.

*Amico KR. Percent total attrition: A poor metric for study rigor in hosted intervention designs. American Journal of Public Health. 2009; 99(9):1567.
