# Supplementary material for: School-based sexual health education interventions to prevent STI/HIV in sub-Saharan Africa: a systematic review and meta-analysis
Source: BMC Public Health. 2016 Oct 10;16:1069. doi: 10.1186/s12889-016-3715-4 (PMC5057258; doi:10.1186/s12889-016-3715-4)
Supplement: Additional file 6: — Implementation Details. (DOCX 33 kb) [file 12889_2016_3715_MOESM6_ESM.docx]

**SUPPLEMENTARY FILE 6: Implementation Details.**

| STUDY | IMPLEMENTER(S) | TRAINING OF INSTRUCTORS | MONITORING (INCLUDING WHO DID THE MONITORING AND/OR HOW IT WAS DONE) | FIDELITY | CHALLENGES ENCOUNTERED DURING IMPLEMENTATION |
| --- | --- | --- | --- | --- | --- |
| Aderibigbe and Araoye 2008 | Not Reported (NR) | NR | NR | NR | NR |
| Agha and Rossem 2004 | Peer educators | Yes (by professional peer educators) | NR | NR | NR |
| Ajuwon and Brieger 2007 | Teachers and peer educators | Yes | Yes | NR | NR |
| Arnold et al 2012 | Teachers, principals and peer educators | Yes (when funds are available) | NR | NR | NR |
| Atwood et al 2012 | Health educators | NR | Yes (quality checklists were completed by co-facilitators to assess fidelity and attendance). | NR | NR |
| Brieger et al 2001 | Peer educators | Yes | Yes (project monitored using a management information system and an organizational development and management checklist). | NR | NR |

**SUPPLEMENTARY FILE 6: Implementation Details Cont.**

| STUDY | IMPLEMENTER(S) | TRAINING OF INSTRUCTORS | MONITORING (INCLUDING WHO DID THE MONITORING AND/OR HOW IT WAS DONE) | FIDELITY | CHALLENGES ENCOUNTERED DURING IMPLEMENTATION |
| --- | --- | --- | --- | --- | --- |
| Burnett et al 2011 | Teachers | NR | NR | NR | NR |
| Cowan et al 2010 | School leavers as peer educators and nurse with other staff working in rural clinics. | Yes (peer educators were trained). | Yes (data on study and non-study activities were collected. Team of scientists made regular assessment of fidelity of implementation). | NR | It was initially intended for in-school and out of school youths but due to high school dropout and out migration it was shifted to the community. |
| Cupp et al 2008 | Teachers and peer educators | Yes | NR | NR | NR |
| Denison et al 2012 | Volunteer peer educators | Yes | NR | NR | NR |
| Esere 2008 | NR | NR | NR | NR | NR |
| Fawole et al 1999 | A community physician and teachers. | Yes (teachers were trained) | NR | NR | NR |
| James et al 2005 | NR | NR | NR | NR | NR |
| James et al 2006 | Teachers | Yes | Yes | Not all schools delivered the whole intervention as designed. | NR |

**SUPPLEMENTARY FILE 6: Implementation Details Cont.**

| STUDY | IMPLEMENTER(S) | TRAINING OF INSTRUCTORS | MONITORING (INCLUDING WHO DID THE MONITORING AND/OR HOW IT WAS DONE) | FIDELITY | CHALLENGES ENCOUNTERED DURING IMPLEMENTATION |
| --- | --- | --- | --- | --- | --- |
| Jemmott et al 2015 | Co-facilitators (who had worked as teachers or had previously taught HIV education) | Yes | NR | NR | NR |
| Karnell et al 2006 | Teachers and peer educators | Yes | By periodic supervision by the research team and teachers are given form to complete after each lesson. | Yes “although the pace of teachers' implementation of program varied, all teachers delivered the full curriculum in the prescribed time”. | NR |
| Mason-Jones et al 2011 | Peer educators | Yes | NR | NR | NR |
| Mason-Jones et al 2013 | NR | NR | NR | NR | NR |

**SUPPLEMENTARY FILE 6: Implementation Details Cont.**

| STUDY | IMPLEMENTER(S) | TRAINING OF INSTRUCTORS | MONITORING (INCLUDING WHO DID THE MONITORING AND/OR HOW IT WAS DONE) | FIDELITY | CHALLENGES ENCOUNTERED DURING IMPLEMENTATION |
| --- | --- | --- | --- | --- | --- |
| Mathews et al 2012 | Teachers | Yes | Yes | Not all students received the whole lesson as designed, some teachers do not implement condom demonstration lessons and many of the teachers replaced the skills-based activities such as role plays and group work due to the large sizes of the class. However, other aspects of implementation fidelity were judged as acceptable. | Some teachers were reluctant to implement lessons on condom use. |

**SUPPLEMENTARY FILE 6: Implementation Details Cont.**

| STUDY | IMPLEMENTER(S) | TRAINING OF INSTRUCTORS | MONITORING (INCLUDING WHO DID THE MONITORING AND/OR HOW T WAS DONE) | FIDELITY | CHALLENGES ENCOUNTERED DURING IMPLEMENTATION |
| --- | --- | --- | --- | --- | --- |
| Maticka-Tyndale et al 2007 | Teachers and peer supporters | Yes | Yes, done by quality assurance officers using ‘teacher-implementation’ and ‘pupil implementation’ scale. | NR | NR |
| Mba et al 2007 | Researcher | NR | NR | NR | NR |
| Menna et al 2015 | Peer educators | Yes | Supportive supervision was done by the principal investigator in collaboration with the respective directors and/or deputy directors of the schools to monitor the effectiveness of the peer education program. | NR | Nature of school programs and scarcity of required resources; lack of free time and money; and lack of motivation among peer educators due to failure to positively address their request to pay for their transportation and refreshment were some of the challenges encountered. |
| Michielsen et al 2012 | Peer educators | Yes | Yes, by coordinators from the organizers. | No | Internal problems in the organisation led to reduce training of peer educators at second half of the intervention and subsequently reduced activities. |

**SUPPLEMENTARY FILE 6: Implementation Details Cont.**

| STUDY | IMPLEMENTER(S) | TRAINING OF INSTRUCTORS | MONITORING (INCLUDING WHO THE MONITORING AND/OR HOW IT WAS DONE) | FIDELITY | CHALLENGES ENCOUNTERED DURING IMPLEMENTATION |
| --- | --- | --- | --- | --- | --- |
| Okonofua et al 2003 | Peer educators delivered the school component of the intervention | Yes | NR | NR | NR |
| Rijsdijk et al 2011 | Peer educators and teachers | Yes | Yes | Not all school delivered all the lesson or use manual as designed. | Poor availability of computers made some schools to deliver the intervention using printed materials. |
| Ross et al 2007 | Teachers, peer educators, condom promoters/distributors | NR | Yes (by government workers and staff member from African Medical Research Foundation). | Yes "…was implemented well and achieved high coverage." | NR |
| Stanton et al 1998 | Volunteer teacher or out of school youth (student teacher or a youth who had completed grade 12) | Yes | Yes (facilitators complete evaluation questionnaire and observers visited class from time to time). | NR | NR |
| Taylor et al 2014 | Trained young male and female facilitators | Yes | NR | NR | NR |

**SUPPLEMENTARY FILE 6: Implementation Details Cont.**

| STUDY | IMPLEMENTER(S) | TRAINING OF INSTRUCTORS | MONITORING (INCLUDING WHO THE MONITORING AND/OR HOW IT WAS DONE) | FIDELITY | CHALLENGES ENCOUNTERED DURING IMPLEMENTATION |
| --- | --- | --- | --- | --- | --- |
| Tibbits et al 2011 | Teachers and youth development specialists. | Yes | NR | NR | NR |
| Van der Maas and Otte 2008 | Trainers and peer educators | Yes | NR | NR | NR |
| Ybarra et al 2013 | Self-administered but supervised by research assistants | NR | NR | Few modifications due to timing of the program were made. | NR |

NR= Not reported.
