# Supplementary material for: School-based sexual health education interventions to prevent STI/HIV in sub-Saharan Africa: a systematic review and meta-analysis
Source: BMC Public Health. 2016 Oct 10;16:1069. doi: 10.1186/s12889-016-3715-4 (PMC5057258; doi:10.1186/s12889-016-3715-4)
Supplement: Additional file 7: — Forest Plots for Sensitivity Analyses. (DOCX 96 kb) [file 12889_2016_3715_MOESM7_ESM.docx]

**SUPPLEMENTARY FILE 7: Forest Plots for Sensitivity Analyses.**

| 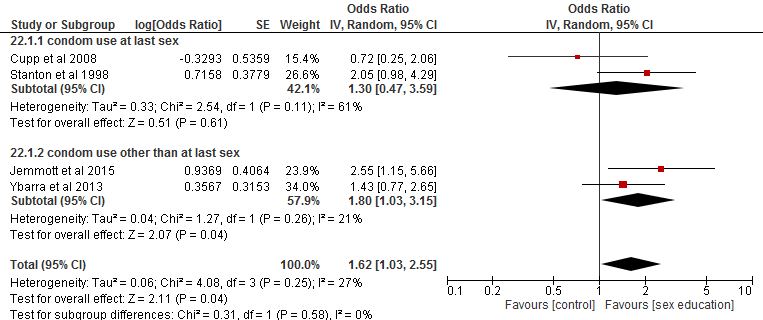   1. Measures of condom use for short follow-up period (RCTs). |
| --- |
| 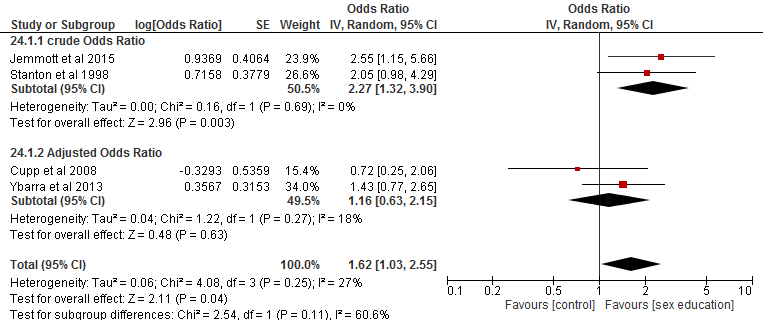   1. Crude vs adjusted Odds Ratios for short-term follow-up period (RCTS). |

**SUPPLEMENTARY FILE 7: Sensitivity Analyses Cont.**

| 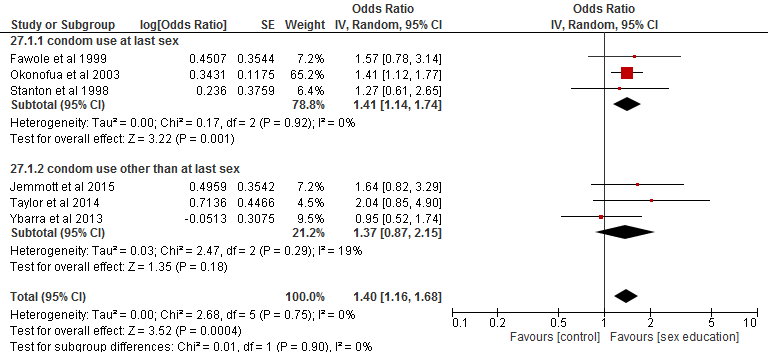   1. Measures of condom use for intermediate follow-up period (RCTs). |
| --- |
| 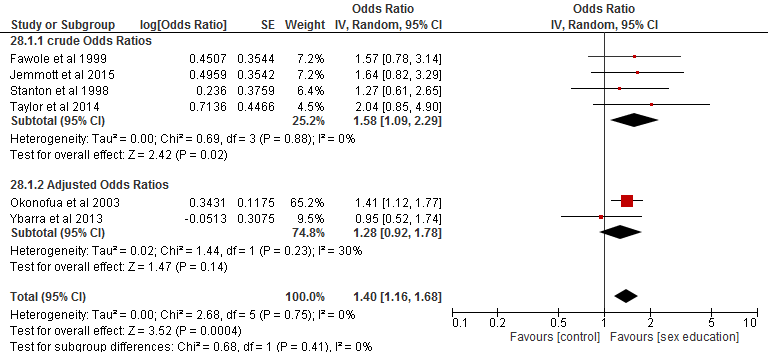   1. Adjusted vs crude odds ratio for intermediate follow-up (RCTs). |

**SUPPLEMENTARY FILE 7: Sensitivity Analyses Cont.**

| 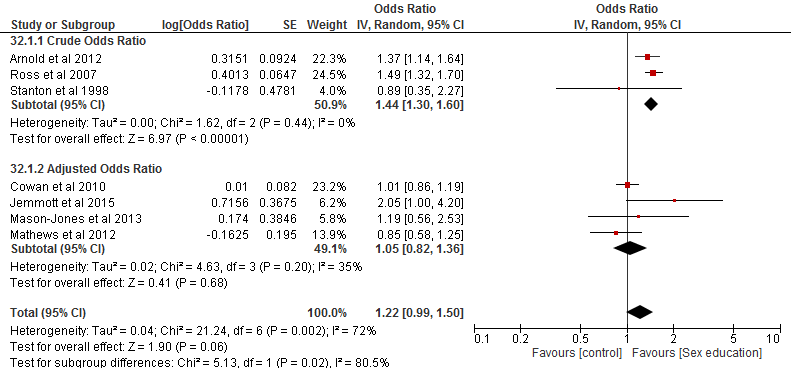   1. Adjusted vs crude odds ratios for long term follow-up (RCTs). |
| --- |
